# Photoisomerization Dynamics of Azo-Escitalopram Using Surface Hopping and a Semiempirical Method

**DOI:** 10.1021/acs.jpcb.4c06924

**Published:** 2024-12-21

**Authors:** Hans Georg Gallmetzer, Eduarda Sangiogo Gil, Leticia González

**Affiliations:** † Doctoral School in Chemistry (DoSChem), University of Vienna, Währinger Str. 42, 1090 Vienna, Austria; ‡ Institute of Theoretical Chemistry, Faculty of Chemistry, University of Vienna, Währinger Str. 17, 1090 Vienna, Austria; § Vienna Research Platform in Accelerating Photoreaction Discovery, University of Vienna, Währinger Str. 17, 1090 Vienna, Austria

## Abstract

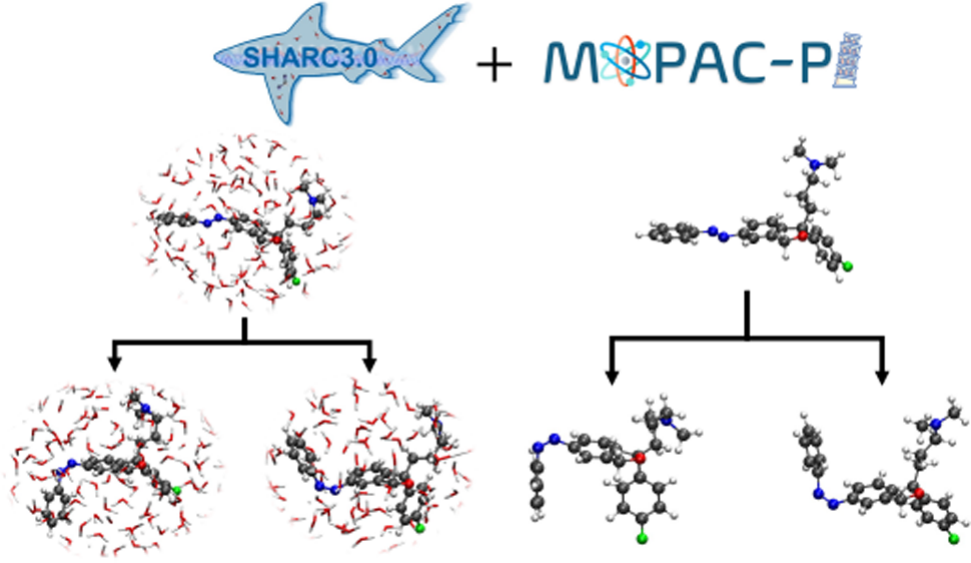

The photoisomerization dynamics of azo-escitalopram, a synthetic photoswitchable inhibitor of the human serotonin transporter, is investigated in both gas-phase and water. We use the trajectory surface hopping method—as implemented in SHARC—interfaced with the floating occupation molecular orbital–configuration interaction semiempirical method to calculate on-the-fly energies, forces, and couplings. The inclusion of explicit water molecules is enabled using an electrostatic quantum mechanics/molecular mechanics framework. We find that the photoisomerization quantum yield of trans-azo-escitalopram is wavelength- and environment-dependent, with *n* → π* excitation yielding higher quantum yields than π → π* excitation. Additionally, we observe the formation of two distinct cis-isomers in the photoisomerization from the most thermodynamically stable trans-isomer, with formation rates influenced by both the excitation window and the surrounding environment. We predict longer excited-state lifetimes than those reported for azobenzene, suggesting that the escitalopram moiety contributes to prolonged lifetimes and slower torsional motions.

## Introduction

**Figure 1:**
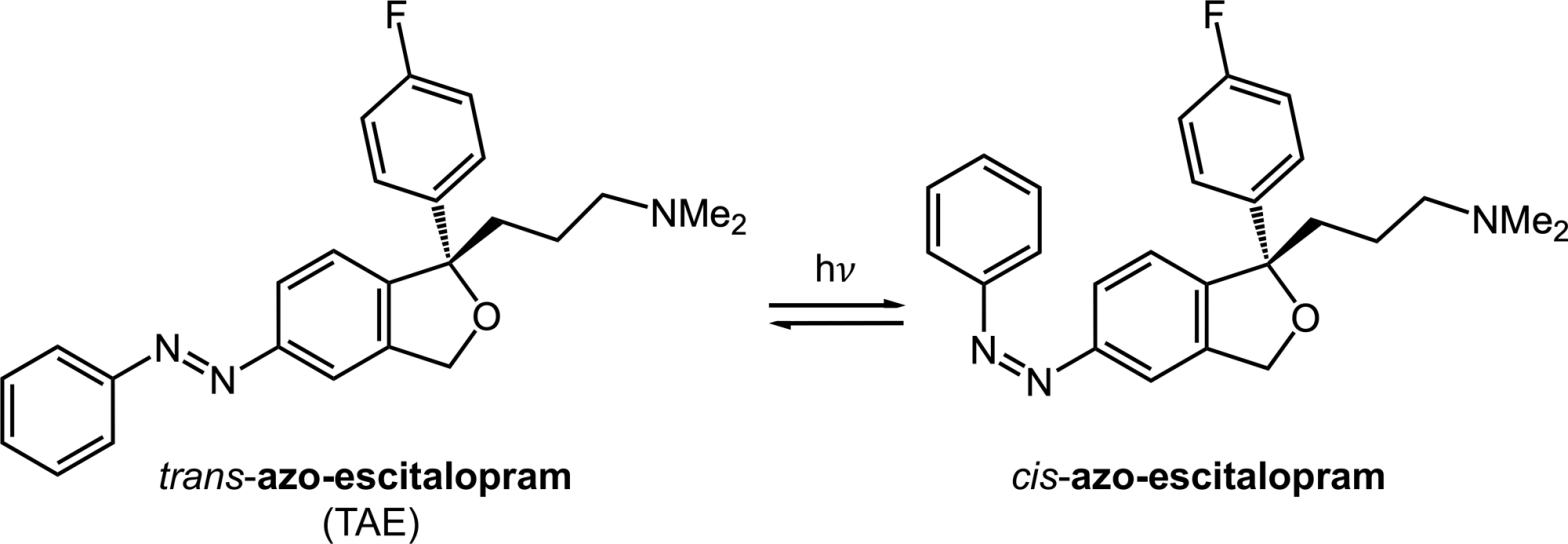
Lewis structure of the two isomers of azo-escitalopram.

The photoswitchable properties of azobenzene have garnered major interest due to their potential applications as molecular switches in fields as diverse as molecular machines, materials science, and biochemistry.^1−7^ Azo-escitalopram (see Figure 1) is a synthetic photoswitchable azobenzene derivative independently investigated by Cheng et al.^8^ as well as Dreier.^9^ It combines the azobenzene core-switchable unit with escitalopram—the (S)-enantiomer form of citalopram, which belongs to the family of selective serotonin reuptake inhibitors (SSRI) of the human serotonin transporter. SSRIs work by increasing the extracellular levels of serotonin in the brain. They do this by inhibiting the reuptake of serotonin into presynaptic neurons, which enhances the concentration of serotonin in the synaptic cleft. This increased availability improves neurotransmission and helps alleviate symptoms of depression over time.^10,11^ Dysregulated serotonin levels are associated with conditions such as depression, attention deficit hyperactivity disorder, epilepsy, decreased libido, and narcolepsy,^12−14^ making SSRIs a commonly prescribed treatment for depression and related mood disorders.

The molecular mechanisms underlying SSRIs’ effects in different brain regions remain elusive. However, photoswitchable SSRIs, such as azo-escitalopram, offer a valuable tool for exploring the spatial and temporal effects of drugs in the brain, thereby enhancing our understanding of their underlying mechanisms and potential therapeutic applications.^8,15^ As an azobenzene derivative, trans-azo-escitalopram (TAE)—the thermodynamically most stable form—can be reversibly photoisomerized to the less stable cis-configuration (Figure 1) using light of different wavelengths.^8^ The interactions of TAE with the human serotonin transporter have already been investigated by ground state molecular dynamics simulations.^16^ Despite its relevance, the photoisomerization reaction has not yet been investigated at the molecular level. However, it is important to understand the structural changes that occur during the photoisomerization, as they alter the molecule’s polarity and shape, thus influencing its biological activity.^17^

In contrast, the excited-state properties of the parent azobenzene have been extensively studied using both experimental and computational methods, including stationary calculations and nonadiabatic dynamics approaches, across various environments.^18−31^ It is well-known that the ultraviolet spectrum of azobenzene shows two main absorption bands: a lower-energy band corresponding to the *n* → π* transition and a higher-energy band associated with the π → π* transition.^32,33^ Notably, the quantum yields of azobenzene photoisomerization vary depending on the wavelength of the incident light.^34−41^

Given the large molecular size of TAE and related SSRIs, the direct application of ab initio electronic structure methods to study its excited-state dynamics presents significant computational challenges. In particular, isomerization reactions involve the formation of radical pairs that are not correctly described by monoconfigurational methods; instead, multireference techniques are mandatory.^42^ An alternative to costly ab initio electronic structure methodology is the use of semiempirical quantum chemical methods, which offer a favorable balance between accuracy and computational efficiency, particularly for organic molecules.^43^ Semiempirical methods streamline the calculation of molecular properties by incorporating approximations, such as minimal basis sets, and by either neglecting or empirically approximating the costly two-electron integrals. Different flavors vary in the level of approximation and the types of empirical data used to parametrize the Hamiltonian, thereby allowing for faster electronic structure calculations and enabling the study of larger systems.^44,45^

The modified neglect of differential overlap (MNDO) family of methods—such as AM1, PMx, and OMx—has been extensively employed in the molecular simulation of organic compounds.^43,46−49^ Particularly relevant for this work is the configuration interaction scheme based on floating occupation molecular orbitals (FOMO–CI),^50,51^ a semiempirical multirefence scheme that has been shown to be suitable for describing the photodynamics of azobenzene and its derivatives across a variety of conditions, including gas-phase, solution, self-assembled monolayers, and biological environments.^22,24−26,28,52−54^

The aim of this paper is to employ the semiempirical FOMO–CI method in combination with the Tully’s fewest switches trajectory surface-hopping algorithm^55,56^ to investigate the photodynamics of TAE, both in the gas-phase and in water. To this end, we have developed an interface between our molecular dynamics package SHARC^57,58^ and the MOPAC-PI program,^59^ thereby enabling surface hopping dynamics with a semiempirical method also in the framework of hybrid quantum mechanics/molecular mechanics (QM/MM)^60,61^ simulations. For both gas-phase and water, we excite TAE at two different wavelengths, corresponding to the electronic *n* → π* and π → π* excitations, to investigate their dynamical response.

The remainder of this paper is organized as follows. First, we describe the implementation of the interface between the semiempirical package with the excited state molecular dynamics suite. We then present the results, beginning with the absorption spectra of TAE in gas-phase and in solution, followed by an analysis of the resulting excited-state dynamics.

## Methods

### Implementation of SHARC Excited State Dynamics Using Semiempirical FOMO–CI

The time-evolution of a nonrelativistic molecular system is governed by the time-dependent Schrödinger equation:
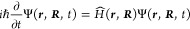
where Ψ(***r***, ***R***, *t*) is the molecular wave function, depending on the electronic coordinates *r*, nuclear coordinates ***R***, and time *t*. The molecular Hamiltonian *Ĥ*(***r***, ***R***) incorporates the kinetic energy of the nuclei and electrons, along with the nuclear–nuclear, electron–electron, and nuclear-electron interactions. Solving this equation for complex molecular systems is highly nontrivial due to the nonlocal nature of quantum mechanics and the computational effort, which grows exponentially with the number of degrees of freedom.

A practical and widely used approximation to simulate excited state dynamics is to treat the nuclei classically while keeping the electrons quantum mechanical. This approach works well when quantum effects (such as tunneling and interference) are negligible, and when the energy differences between quantum levels are small compared to the kinetic energy of the nuclei. Since nuclei are much heavier than electrons, their quantum level spacing is generally smaller. Thus, treating the nuclei classically is reasonable, while electrons are treated quantum mechanically.

In the surface hopping method,^62−64^ it is assumed that for most of the dynamics, the nuclei move adiabatically on a single potential energy surface (PES). Nonadiabatic transitions—where the system switches between different PESs—are modeled as “hops” that occur briefly in small regions of the configuration space. Surface hopping requires the simulation of many independent trajectories, each following a classical path, and the fraction of these trajectories on each PES represents the population of different electronic states. For the nuclear motion, the time evolution of the classical degrees of freedom is determined by integrating Newton’s equations, with forces calculated from the PES on which the system currently resides. One major advantage of trajectory surface hopping is that electronic properties, such as energies, gradients, and nonadiabatic couplings, are calculated *on-the-fly*, meaning that no prior knowledge of the PESs or coupling terms is required. At each time step, an electronic structure calculation is required to solve the electronic Schrödinger equation and update the positions and velocities of the nuclei. The choice of this method is critical, as it directly impacts both the accuracy and feasibility of the simulation.^65^ The quality of the PESs, which determine the dynamics, depends on the accuracy of the electronic structure calculations, and errors in the PES can lead to incorrect predictions of system behavior. The computational cost of trajectory surface hopping simulations is thus mostly dictated by the underlying electronic structure calculations, so it is essential to find a balance between accuracy and efficiency.^66^

The employed electronic structure method must therefore meet several key requirements. It must provide reasonable solutions for both, ground and excited states, describe all nuclear configurations encountered during the trajectory on the same footing (including processes like bond breaking and state degeneracies), and be computationally feasible, as the method is called at every time step during the dynamics. Since nuclear time steps can be as small as 0.1 fs, a simulation of 1 ps requires at least 10,000 electronic structure calculations, which can be computationally quite expensive. While some ab initio methods meet the first two criteria, they are often computationally too expensive to simulate dynamics of large systems. It is in this regard that semiempirical methods offer a viable alternative for balancing accuracy and computational cost in medium to large systems.

In this work, we will employ the semiempirical FOMO–CI method^50,51^ implemented in the MOPAC-PI program.^59^ In the FOMO–CI, the electronic wave functions are based on configuration interaction (CI) that uses molecular orbitals obtained from a single-determinant self-consistent field calculation. A key feature is that these molecular orbitals have fractional or “floating” occupation numbers, meaning that the number of electrons in each orbital is not fixed at 0 or 2 but fractional. The orbitals are divided into inactive, active, and virtual, but only the active ones have fractional or ”floating” occupations. The occupations can be either predefined or determined dynamically based on orbital energies, providing flexibility in handling bond-breaking and twisting processes—as it happens in photoisomerization processes. Given its demonstrated ability to accurately describe nonadiabatic dynamics in various systems, including mechanisms such as photoisomerizations, excitonic transfer, and singlet fission,^20,26−28,30,44,52^ this method has been chosen to simulate surface hopping dynamics using semiempirical methods. Accordingly, we combined the molecular dynamics program SHARC (Surface Hopping including ARbitrary Couplings)^57,58^ with MOPAC-PI.^59^

**Figure 2:**
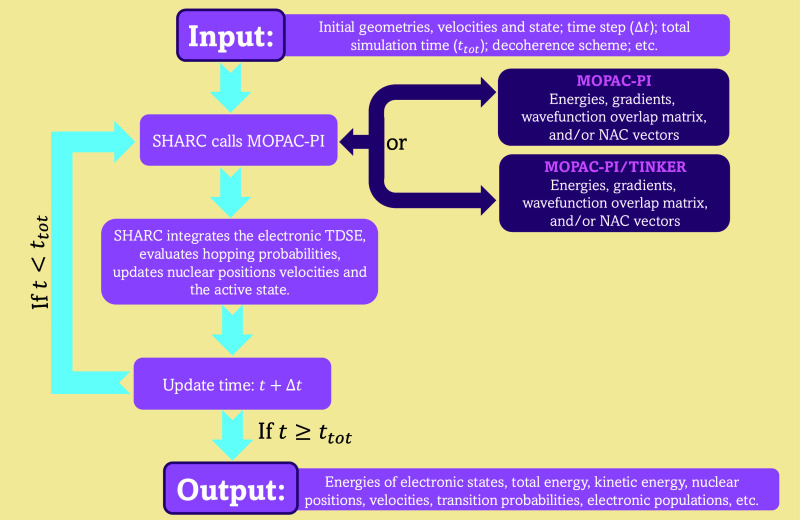
Workflow for performing surface hopping dynamics with SHARC and MOPAC-PI. The algorithm starts by initializing input parameters, such as initial geometry, velocities, and simulation time. SHARC then calls MOPAC-PI (or MOPAC-PI/TINKER in the case of a QM/MM scheme), which computes electronic energies, gradients, the wave function overlap matrix, and/or nonadiabatic coupling (NAC) vectors. SHARC reads these outputs and uses them to integrate the time-dependent Schrödinger equation (TDSE), evaluate hopping probabilities, and update nuclear positions and velocities. The simulation time is incremented by discrete steps (Δ*t*) until the total simulation time is reached. The final output includes quantities such as electronic and total energies, nuclear positions, velocities, transition probabilities, and electronic populations.

The workflow used to perform surface hopping dynamics using SHARC and MOPAC-PI is schematically represented in Figure 2. In this setup, MOPAC-PI (or MOPAC-PI/TINKER, for a QM/MM scheme) computes the electronic energies, energy gradients, and either the wave function overlap matrix or the nonadiabatic coupling vectors. These outputs are then utilized by SHARC to integrate the electronic time-dependent Schrödinger equation, calculate hopping probabilities, and update the nuclear positions and velocities accordingly. The propagation of electronic coefficients can be achieved using either nonadiabatic coupling vectors or the local diabatization algorithm.^51,67^ The latter method avoids the explicit calculation of nonadiabatic coupling vectors and instead relies on the wave function overlap matrix between consecutive time steps. In the current implementation, we leverage MOPAC-PI’s built-in functionality to directly compute the overlap matrix, bypassing the approach implemented in SHARC by default that calls the WFoverlap^68^ program for this calculation. We opted for the local diabatization scheme because nonadiabatic coupling vectors tend to fluctuate rapidly, necessitating very small time steps to maintain numerical stability, especially in regions of strong interaction, such as avoided crossings and conical intersections. Moreover, methods that explicitly use nonadiabatic coupling vectors for propagating electronic coefficients often fail to accurately handle “trivial crossings” between uncoupled states, regardless of time step size. By employing local diabatization, we circumvent these challenges and enable the use of larger time steps, as the (expensive) calculation of the nonadiabatic coupling vector is not needed. It should be noted, however, that nonadiabatic coupling vectors can be calculated with MOPAC-PI and used by SHARC, if desired. Such vectors can be required not only for propagating electronic coefficients in SHARC^69,70^ but also for rescaling the momentum after a hop,^71^ or be used in combination with other mixed quantum-classical dynamics methods available within the SHARC suite.^57^

In order to perform excited state simulations in the presence of an explicit environment—water in this case—the dynamics is carried out in a hybrid QM/MM framework.^60,61^ Here, the QM and MM subsystems interact through electrostatic embedding, where the electrostatic potential generated by the classical MM point charges (from atoms in the MM region) is explicitly incorporated into the QM Hamiltonian. This allows the QM system to experience the electrostatic field of the MM region, meaning that the MM charges influence the electronic structure of the QM subsystem by directly interacting with the QM electron density and nuclei.^72^ Since MOPAC-PI already includes an internal interface with the TINKER package,^73^ this can conveniently provide energies, gradients, and overlap matrices within the QM/MM scheme. In passing we note that, although polarizable embedding is implemented in TINKER, extending polarizable models to nonadiabatic dynamics is challenging due to the nonlinearity introduced into the Hamiltonian by the polarization degrees of freedom, which depend on the QM charge density. This results in state-specific nonlinear Hamiltonians, potentially causing electronic states to lose orthogonality. Therefore, implementing a state-specific environment model becomes nontrivial in the context of surface hopping dynamics. For this reason, electrostatic embedding is often preferred for treating the MM environment in this context. Polarizable embedding has been implemented within the time-dependent density functional theory framework by Bondanza et al.^74^ but extending its formulation and implementation to nonadiabatic dynamics is a significant undertaking.

### Computational Details

The electronic properties necessary to propagate the nuclear dynamics in both the gas-phase and solution—energies, energy gradients, and electronic wave functions—are computed using the FOMO–CI method with a semiempirical AM1 Hamiltonian, reparameterized for azobenzene by Cusati et al.^23^ In both environments, we considered an active space of six electrons in four orbitals. These correspond to one of the nonbonding orbital localized on the nitrogens of the azobenzene core unit, the two highest lying π orbitals, and one π* orbital (Section S1).

The QM/MM calculations were performed using the OPLS force field,^75^ with water modeled by the SPC-Fw water model,^76^ as implemented in the Tinker (Version 8.5) package.^73^ The interaction between the QM and MM regions is achieved via electrostatic embedding, as explained above. To simulate solvation effects, a sphere consisting of 2000 water molecules was considered.

The initial conditions (geometries and velocities) for the excited-state dynamics in gas-phase and in water were sampled from a classical Boltzmann distribution obtained from a QM/MM ground-state thermal trajectory at 300 K. The thermalization was maintained using a Langevin thermostat^77^ with a friction coefficient of 0.02 fs^–1^. The thermal dynamics were propagated with a nuclear time step of 0.5 fs, and simulations were run for 20 ps in the gas-phase and 25 ps in water. In both the gas-phase and water, the system was allowed to reach equilibrium, and the initial geometries and velocities were sampled from the last 15 ps. The results of these thermal simulations, which demonstrate that the system is equilibrated, are presented in Section S2. Also noteworthy is that the hydrogen bond distances of the five heteroatoms of TAE in water reached a stable equilibrium before 10 ps (Figure S6).

Four sets of excited-state simulations were carried out for TAE: (i) 112 trajectories in gas-phase starting from the *n* → π* state; (ii) 254 trajectories in gas-phase starting from the π → π* state; (iii) 130 trajectories in water starting from the *n* → π* state; and (iv) 194 trajectories in water starting from in the π → π* state. We note that at least 100 trajectories are typically sufficient to model processes occurring in at least 5% of trajectories. For example, if we consider an ensemble of 100 trajectories for a process occurring in 5% of the trajectories, the margin of error for its probability is ±4%, which is adequate for capturing major trends.^78^ Since we used more than 100 trajectories for all four sets of trajectories, and the photoisomerization quantum yield and other pathways occur with a probability greater than 5%, we expect this number to be sufficient for obtaining representative results. Table 1 summarizes the four sets of trajectories, including the total number of trajectories run (*N*_total_) and the number of trajectories considered in the analysis (*N*_Traj_). Trajectories that failed to conserve total energy during the dynamics (with energy variations exceeding 0.5 eV) were excluded from the analysis.

**Table 1:** Number of Simulated Trajectories for TAE under Different Conditions, Including the Actual Number of Trajectories Run (*N*_total_) and the Number of Trajectories Analyzed (*N*_Traj_)^a^

	excitation	*N*_Traj_	*N*_total_
gas-phase	*n* → π*	112	112
	π → π*	207	254
water	*n* → π*	125	130
	π → π*	185	194

a Trajectories with total energy variations exceeding 0.5 eV were excluded from the analysis.

The sampling process accounted for radiative (dipole) transition probabilities in each window. Trajectories where the total energy changed by more than |0.5| eV during the excited-state dynamics—primarily due to changes in the orbitals of the active space leading to inconsistent PES—were excluded from the analysis. In vacuum, the excited-state trajectories were propagated for 6 ps using a nuclear time step of 0.1 fs. In water, propagation was stopped after 3 ps and a time step of 0.2 fs was employed. However, trajectories that reached the ground state and remained there for at least 500 fs were terminated earlier.

As mentioned above, the local diabatization algorithm^51,67^ was used for integrating the electronic time-dependent Schrödinger equation. The Granucci-Persico energy-based decoherence correction,^79^ with a standard parameter of 0.1 au, was applied. Nuclear velocities were rescaled after a hop in the direction of the nuclear momentum. This is necessary to preserve the total energy during the dynamics, as the potential energy of the electronic state after the hopping is typically different from that before the hopping.

For trajectories starting in the *S*_1_ (*n* → π*) state, the time evolution of the *S*_1_ classical population (*P*_*n*→π*_) is modeled by a delayed exponential function:
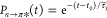
1where *t*_0_ represents a delay time, and  is the characteristic time constant. The lifetime of the *n* → π* state (*S*_1_) is given by
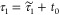
In the case of trajectories initiated in the *S*_2_ state, the lifetimes of both the *S*_1_ (*n* → π*) and *S*_2_ (π → π*) states are determined using a two-step irreversible kinetic model. The population dynamics for the *n* → π* and π → π* states are described by
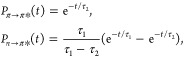
2where τ_2_ is the lifetime of the π → π* state, and τ_1_ is the lifetime of the *n* → π* state. In this model, the *P*_π→π*_(*t*) population decays exponentially with a characteristic time constant τ_2_, after which the *P*_*n*→π*_(*t*) population rises due to transfer from the π → π* state and then decays exponentially with time constant τ_1_. Importantly, in both sets of trajectories—whether starting in the *S*_1_ (*n* → π*) state or the *S*_2_ (π → π*) state—the parameter τ_1_ consistently represents the lifetime associated with the decay of the *S*_1_ (*n* → π*) state. This ensures that τ_1_ always reflects the dynamics of the *S*_1_ state, irrespective of the initial state of the system. The fitting procedure for the trajectories starting in the *S*_2_ state is carried out in two steps: first, τ_2_ is determined by fitting the population decay of *P*_π→π*_(*t*). Once τ_2_ is fixed, τ_1_ is then obtained by fitting the population dynamics of *P*_*n*→π*_(*t*).

**Figure 3:**
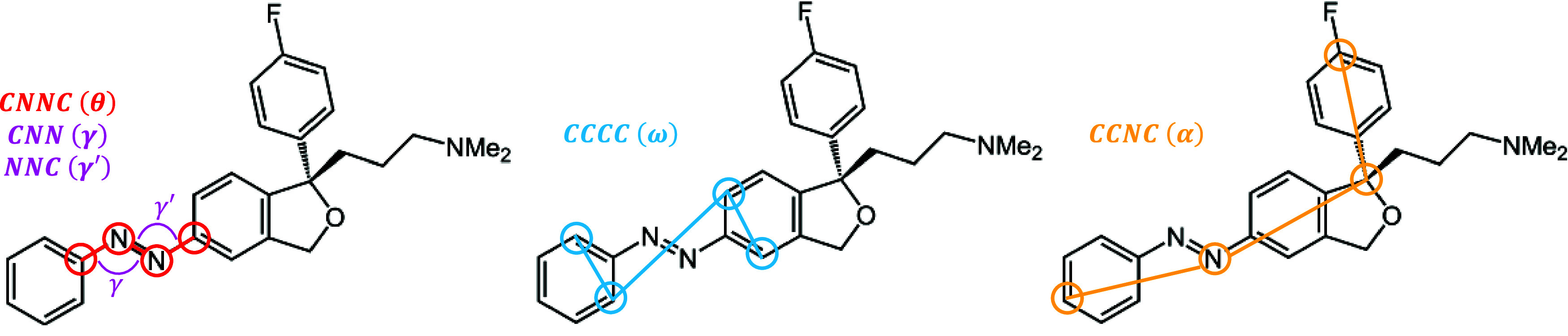
Relevant internal coordinates of TAE: the CNNC dihedral (θ), CNN and NNC angles (γ and γ′), CCCC dihedral (ω), and CCNC dihedral (α).

Partial photoisomerization quantum yields (Φ) are calculated with the trajectories that reach the ground state, irrespective of when this occurs—whether at the end of simulation or earlier (and stay at least during 500 fs). Trajectories are labeled as cis or trans if the CNNC dihedral angle θ (see definition in Figure 3) is less than 30° or larger than 150°, respectively. The quantum yield Φ is defined as the ratio of cis trajectories to the total number of trajectories in the ground state,
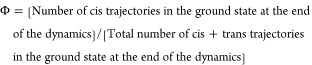
3

As it will be shown in the Results section, TAE can evolve into two possible cis-conformers, labeled as I and II and defined according to the CCNC dihedral angle α (Figure 3), if this is less than 45° (I) or larger than 135° (II). Thus, we also introduce ϕ^*x*^, as the ratio of trajectories in the *x* (*x* = I, II) cis conformation, calculated as,
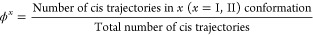
4

All nuclear dynamics were carried out using the SHARC package,^57^ with electronic properties extracted from the MOPAC-PI program.^59^

### Energy Conservation

Several tests were conducted to validate our semiempirical FOMO–CI–SHARC implementation, paying particular attention to investigate whether the total energy is conserved during the dynamics. Energy conservation is essential in dynamical simulations to ensure physical accuracy, numerical stability, reliable estimations of rates and long-term reliability of the simulation results. Therefore, we monitored the total energy and the variations in the energy gradient throughout the dynamics. In particular, when using CI-methods, the total energy can fluctuate because active states can suffer from orbital switching within the active space during the dynamics. Such behavior is not desirable because it causes changes in both the energy and the corresponding gradients, affecting the underlying mechanism. When the energy gradients exhibit sudden “spikes”, it often indicates that the forces acting on the nuclei, which in surface hopping are the negative of the energy gradients, might become erratic. For this reason, sudden variations in the gradients may easily lead to inaccurate dynamics and should not be ignored.

In our simulations, we set a threshold of 0.5 eV throughout the entire duration of the dynamics (Section S3) to monitor the conservation of total energy. Figures S7 and S8 display the variation in total energy across all trajectories for the simulations initiated in the *n* → π* and π → π* states, respectively. For the trajectories starting from the *n* → π* state (Figure S7), energy was conserved both in gas-phase and water, being exceptionally stable in gas-phase, where total energy fluctuations remained consistently below |0.1| eV. Trajectories exceeding the 0.5 eV threshold were excluded from further analysis. Interestingly, for the trajectories starting in the π → π* state (Figure S8), the energy is better conserved in water than in gas-phase. This is because molecules excited to the π → π* state in gas-phase are higher in energy and face less steric hindrance than those in water, leading to more pronounced structural changes. These changes cause greater fluctuations in the active state orbitals, which likely contribute to less stable total energy conservation.

The robustness of energy conservation was also evaluated against different time step sizes (Section S4). As expected, larger time steps result in greater energy fluctuations, even if the simulations remain numerically stable across all time step sizes investigated.

## Results and Discussion

### Ground State Thermal Dynamics and Absorption Spectra

**Figure 4:**
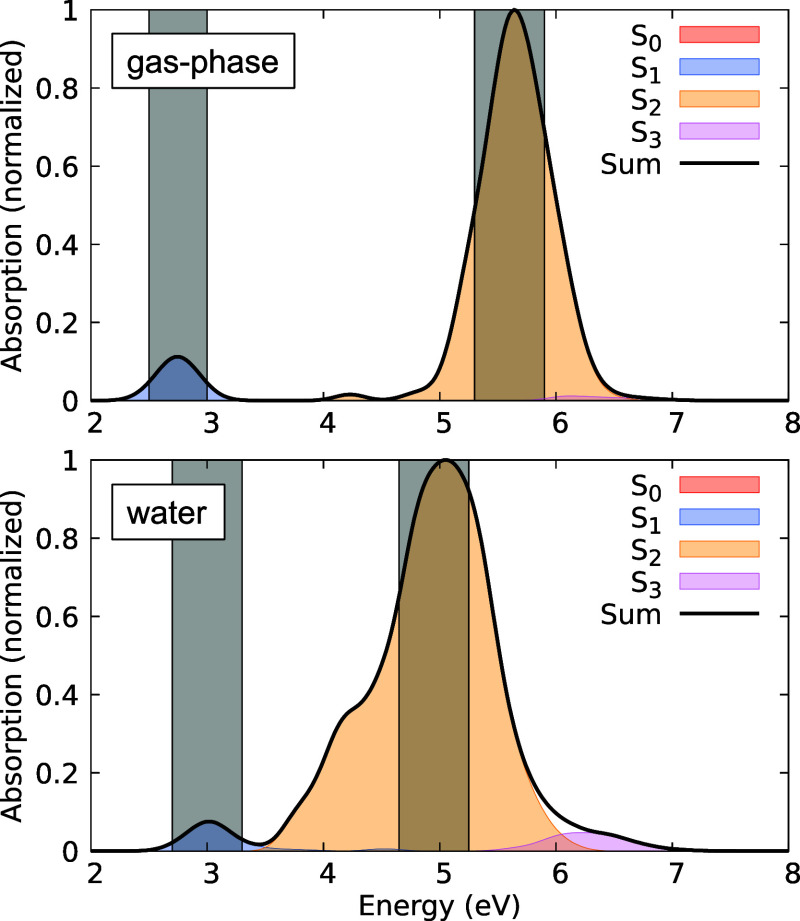
Absorption spectrum of TAE in gas-phase (top) and in water (bottom) calculated from 857 geometries. Filled colors indicate the contributions from each adiabatic state as indicated and the black line represents the total spectrum. Gray areas are the excitation energy windows used for the subsequent dynamics.

Figure 4 shows the computed absorption spectra of the thermodynamically most stable TAE conformation in gas-phase and water, as derived from the thermalization trajectories. Both spectra exhibit two main bands: a lower-intensity band at lower energy corresponding to the *n* → π* transition (*S*_1_ state), and a brighter, higher-energy band associated with the π → π* transition (*S*_2_ state). However, the difference between the two environments on the absorption spectrum is evident. In gas-phase, the *S*_1_ state appears at 2.75 eV, whereas in water, it is blue-shifted by 0.27 to 3.02 eV. Conversely, the *S*_2_ state, located at 5.65 eV in gas-phase, experiences a red-shift of 0.60 eV, appearing at 5.05 eV in water. Additionally, a small shoulder observed at 4.23 eV in the gas-phase spectrum becomes significantly more pronounced in water.

Typically, π → π* states in water are lower in energy compared to the gas-phase, as it is the case here. This is because water, being a polar solvent, tends to stabilize both the ground and excited states due to dipole interactions, but the effect is stronger in the excited state, decreasing the excitation energy. By contrast, the *n* → π* states are generally higher in energy compared to the gas-phase because the antibonding π* orbital does not interact as strongly with the solvent as the lone pair orbitals in the ground state, thereby leading to a larger energy gap.

Cheng and co-workers^8^ measured the absorption spectrum of TAE (in 10% DMSO in PBS solution) and identified two peaks at approximately 410 nm (3.0 eV) and 320 nm (3.9 eV) (Section S5). While the *n* → π* transition aligns well with the experimental data, the π → π* band in our computed water spectrum is noticeably blue-shifted.

In order to rationalize the spectral differences between gas-phase and water, we analyzed the CNNC dihedral (θ), and the CNN and NNC angles (γ and γ′) (see the definitions of the internal coordinates in Figure 3) during the ground state thermalization dynamics, with the aim of identifying geometrical variations that could account for the observed spectral shifts. The time-averaged angles (Figures S4 and S5) do not show pronounced structural differences between the two environments that could explain such large variation in transition energies. We therefore conclude that the changes in the spectrum are mainly due to electrostatic interactions between TAE and water, which can modify orbital energies and, consequently, the excitation energies.

Based on the obtained spectra, four excitation windows for the subsequent excited state simulations have been chosen. In gas-phase, two propagations are carried out. One started from the excitation window set between 2.50 and 3.00 eV, which covers the *n* → π* transition and consists of 112 trajectories initially placed in the *S*_1_ state. The other excitation window was chosen between 5.30 and 5.90 eV to cover the π → π* transition. It contains 254 trajectories in the *S*_2_ state and 1 trajectory in the *S*_3_ state. In water, the two sets of simulations are chosen so that the energetically lower, *n* → π*, excitation window extends from 2.70 to 3.30 eV and yields 130 trajectories in the *S*_1_ state. The higher π → π* band is excited with an excitation window between 4.65 and 5.25 eV, resulting in 194 trajectories.

### Excited State Dynamics

It is well-known that the minimum energy path on the *S*_1_ potential of azobenzene connects the trans and cis geometries to the *S*_1_/*S*_0_ conical intersection, which is located around 95° of torsion in the CNNC (θ) dihedral angle.^32,80,81^ Since TAE incorporates an azobenzene core to the attached escitalopram moiety, it is expected to exhibit a similar photochemical behavior. In isolated trans-azobenzene (TAB), nonadiabatic transitions to *S*_0_ typically occur within this region or slightly earlier, when the CNNC dihedral is between 90° and 120°.^26,27,29^ Because during the relaxation to the ground state, the trans isomer can isomerize into the cis form, the CNNC dihedral and the NNC angles (see Figure 3) are the most important internal coordinates to investigate.

From this point forward, we will refer to trajectories that after reaching the ground state exhibit a cis configuration as *reactive* trajectories, and those that keep the trans configuration as *nonreactive* trajectories.

Photoisomerization quantum yields for TAB in low-viscosity solvents have been measured by various groups,^34−41^ ranging from 0.20 to 0.32 for *n* → π* excitation and 0.09 to 0.16 for π → π* excitation. Likewise, there is a plethora of theoretical studies on TAB’s excited-state dynamics.^18,22,23,26,29^ In contrast, to the best of our knowledge, no reported values for the photoisomerization quantum yield of TAE exist. Our calculated photoisomerization quantum yields (Φ) for the trans-to-cis conversion in both the gas-phase and water are reported in Table 2. We cautiously consider these quantum yields as “partial” to account for the fact that some trajectories are still in the excited state after the end of the simulation-time. Trajectories that decay rapidly tend to predominantly retain the trans configuration upon relaxation to the ground state (nonreactive), whereas slower trajectories are more likely to undergo photoisomerization (reactive). The quantum yield for *n* → π* excitation is approximately 0.25 in the gas-phase and 0.20 in water, both higher than for π → π* excitation, which yielded 0.20 in the gas-phase and 0.15 in water.

**Table 2:** Summary of Results from Four Sets of Excited-State Simulations for TAE, in Both Gas-Phase and Water^a^

	excitation window	Φ^b^	ϕ^(I)^	ϕ^(II)^	*t*_0_	τ_1_	τ_2_
gas-phase	*n* → π* [2.50–3.00 eV]	0.25 ± 0.04	0.67	0.33	250 fs	1939 ± 1 fs	
	π → π* [5.30–5.90 eV]	0.20 ± 0.03	0.52	0.48		1274 ± 1 fs	146 ± 1 fs
water	*n* → π* [2.70–3.30 eV]	0.20 ± 0.04	0.18	0.82	250 fs	2795 ± 3 fs	
	π → π* [4.65–5.25 eV]	0.15 ± 0.03	0.40	0.60		2292 ± 3 fs	26 ± 1 fs

a The table lists excitation energy windows (in eV), photoisomerization quantum yields (Φ) at the end of the simulations, rates of the observed cis isomers (ϕ^(I)^ and ϕ^(II)^), *S*_1_ state lifetime (τ_1_) in fs, *S*_2_ state lifetime (τ_2_) in fs, and delay time (*t*_0_) in fs (see eqs 1 and 2).

b Binomial standard deviation, obtained as .

**Figure 5:**
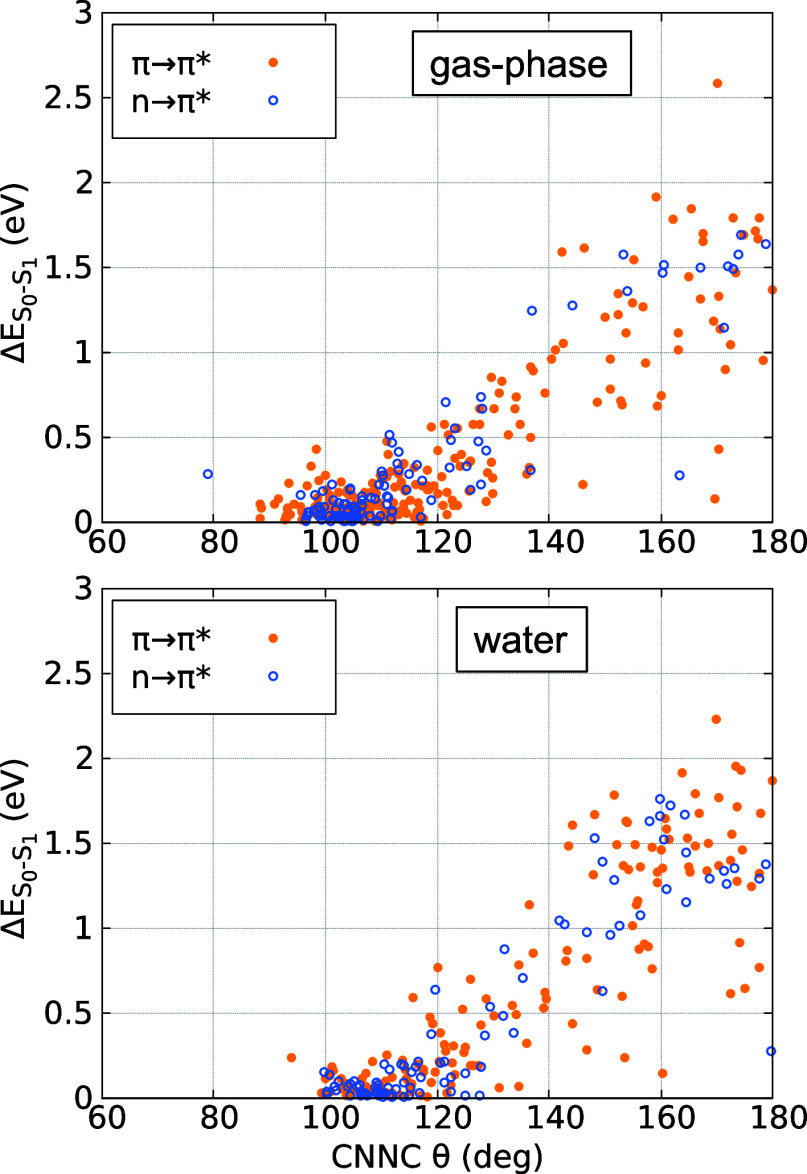
CNNC dihedral angle versus *S*_0_ – *S*_1_ energy differences for the *S*_1_ → *S*_0_ hopping geometries in gas-phase (top) and water (bottom). The π → π* excited trajectories are shown in orange, and *n* → π* excited trajectories in blue.

Figure 5 shows the CNNC dihedral angle versus the *S*_0_ – *S*_1_ energy differences for the *S*_1_ → *S*_0_ hopping geometries in gas-phase (top) and water (bottom) for both excitation windows. We observe that most hopping events occur at small *S*_0_ – *S*_1_ energy differences when the CNNC (θ) dihedral angles are between 90° and 120°. Some trajectories, however, decay at larger dihedrals when the energy differences are also larger. In water, more trajectories hop at large dihedral angles, correlating with the smaller photoisomerization quantum yield compared to gas-phase, especially for π → π* excitation.

**Figure 6:**
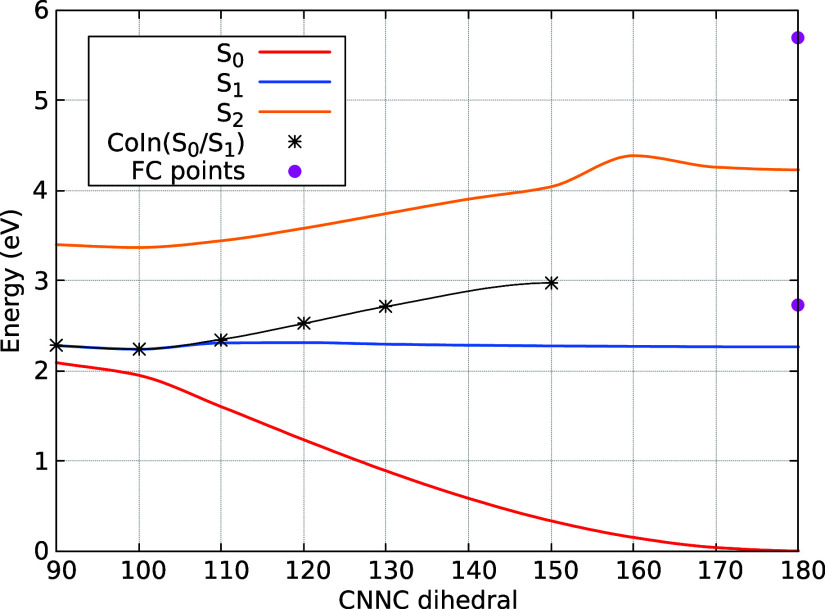
Relaxed potential energy curves of the lowest singlet states of TAE as a function of the CNNC (θ) dihedral (remaining coordinates are optimized for each state). Franck–Condon (FC) points indicated as magenta dots and *S*_0_/*S*_1_ crossing seam as black star symbols.

In order to understand these results better, it is useful to plot the potential energy curves of the first three singlet states along the CNNC dihedral (Figure 6). Also shown in the figure are the Franck–Condon excitation energies of TAE at the ground state equilibrium geometry (magenta points), located at 2.731 eV for the *S*_1_ state (*n* → π* character) and 5.686 eV for the *S*_2_ state (π → π* character). The curves have been optimized for each electronic state, this is why the energies are located below the vertical excitation energies values obtained at the equilibrium geometry. This indicates that the optimized geometries correspond to the minimum energy configurations for each state, which are lower than the Franck–Condon excitation point (the geometry corresponding to the minimum of the ground state) due to the relaxation of the system upon excitation.

The optimized conical intersections between the *S*_0_ and *S*_1_ states at various dihedral angles between 90° and 150° are indicated by black stars.

As shown, the *S*_1_ potential energy curve is relatively flat with a shallow minimum, indicating a gradual energy change as the system approaches the conical intersection region. The minima in the *S*_1_ state at dihedral angles between 90° and 110° align with the optimized crossing seams for those fixed dihedrals, supporting the observation that most hopping geometries occur in this region. However, due to unoptimized crossing seams at larger dihedral angles, it remains uncertain whether the *n* → π* state can approach the crossing seam at these higher angles.

For comparison, studies on TAB have reported a crossing seam where *n* → π* excitations occur at lower energy than the seam at larger dihedral angles, while π → π* excitations occur at higher energies.^26,32,82^ In both reactive and nonreactive *S*_1_ → *S*_0_ internal conversions (where the final product is the cis or trans isomer, respectively), torsion along the CNNC coordinate is required. However, in the unreactive internal conversions, the transition can occur earlier—before reaching the 90° torsional midpoint—especially when more vibrational energy is available, as is typical following π → π* excitation. This explains TAB’s higher photoisomerization quantum yield for *n* → π* excitation, as the π → π* excitation provides enough energy to reach the crossing seam at larger dihedrals.

Figure 5 illustrates that *S*_1_ → *S*_0_ transitions at larger dihedral angles with small energy differences are rare for trajectories starting in the *n* → π* state. This indicates that such transitions are more likely when the π → π* state is initially excited, potentially explaining the higher photoisomerization quantum yield observed for the *n* → π* state compared to the π → π* state. Additionally, the quantum yield is lower in water than in the gas-phase for both excitation windows. However, the proportion of yields between the two excitation windows remains consistent across environments. The reduced yield in water can be attributed to a higher frequency of transitions occurring at larger dihedral angles and with larger energy differences between the *S*_0_ and *S*_1_ states (see Figure 5).

**Figure 7:**
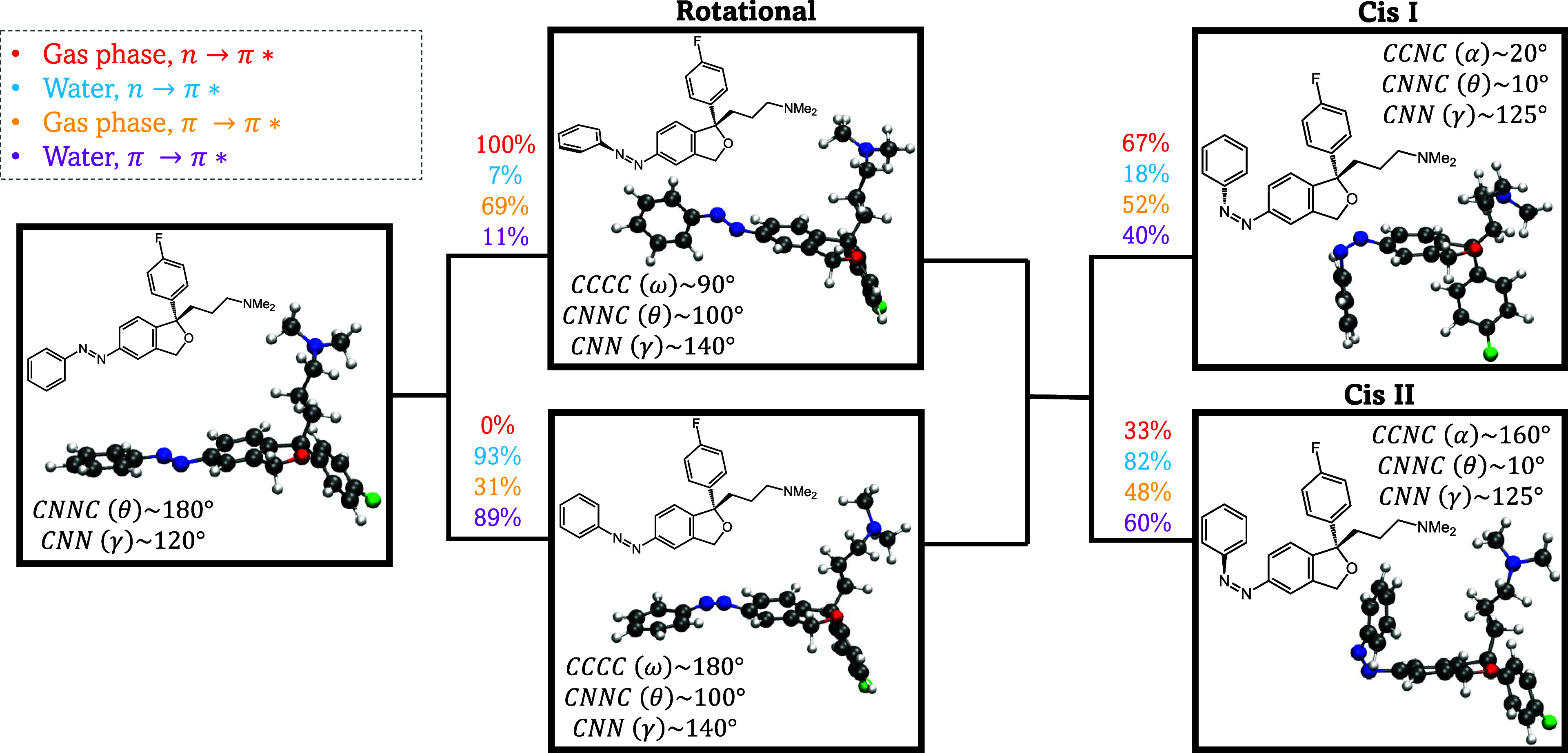
Schematic overview of the reactive trajectories in the photoisomerization dynamics of TAE. The initial configuration is the trans structure, which can access the strong nonadiabatic region through two distinct mechanisms: one is the rotational pathway, involving torsion around the CNNC (θ) dihedral angle of the azo group; the other is the inversion pathway, characterized by in-plane bending of the NNC (γ) angle between the azo group and the carbon of one of the benzene rings. These pathways lead to the formation of two possible cis isomers, labeled as cis I and cis II. The preferred mechanisms and resulting isomers from the four sets of simulations are color-coded: gas-phase and *n* → π* state (red), water and *n* → π* state (blue), gas-phase and π → π* state (orange), and water and π → π* state (purple).

Interestingly, the simulations revealed the formation of two distinct cis isomers, labeled as cis I and cis II in Figure 7. In cis I, the phenyl ring is oriented toward the fluorobenzene group, whereas in cis II, the phenyl group is directed toward the amino group. Table 2 evidences a preference for specific cis-isomers depending on the excitation window in the gas-phase simulations. Upon irradiation, 67% of reactive trajectories excited to the *n* → π* state and 52% to the π → π* state result in the formation of cis-isomer I. In the case of the simulations in water, both excitation windows show a stronger preference for cis-isomer II. Specifically, 82% of trajectories started from the *n* → π* excitation and 60% from the π → π* state result in the formation of cis-isomer II (see Table 2). We also assessed the stability of the two cis conformers. Since conformer II is more stable than I only by 0.0663 kcal mol^–1^, the two conformers can be considered to be isoenergetic.

We investigated whether it is possible to explain the preference for the formation of cis isomers I and II by examining the mechanism of photoisomerization. It is known that TAB undergoes photoisomerization via two distinct pathways, typically known^1,32,80,83^ as rotation and inversion. The rotational pathway involves torsion of the azo group around the CNNC dihedral angle, while the inversion pathway occurs through an in-plane bending of the NNC (γ) angle formed between the azo group and the carbon attached to one of the benzene rings. Importantly, the hopping geometries (i.e., crossing seams) exhibit distinct structures depending on the pathway. In the rotational pathway, the crossing seam structure shows one of the phenyl rings positioned perpendicular to the escitalopram, while in the inversion pathway, the phenyl ring remains relatively planar. Both rotational and inversion mechanisms are illustrated in Figure 7.

By analyzing the geometries of the *S*_0_ → *S*_1_ hopping events, we quantified the number of trajectories following each pathway across different excitation windows and environments. Our objective was to determine whether the preference for specific cis products is related to these hopping pathways. To achieve this, we examined the CCCC (ω) dihedral angle of the reactive trajectories (see Figure 7). The trajectories are then classified based on the CCCC (ω) angle: those between 55° and 125° were assigned to a rotational mechanism, while angles between 145° and 180° corresponded to an inversion mechanism. The ratio of trajectories for each process is summarized in Table 3.

**Table 3:** Quantification of Reactive Trajectories for Rotational and Inversion Mechanisms Based on the CCCC (ω) Dihedral Angle^a^

		rotational	inversion
gas-phase	*n* → π*	1.00	0.00
	π → π*	0.69	0.31
water	*n* → π*	0.07	0.93
	π → π*	0.11	0.89

a The table presents the ratio of trajectories categorized into two groups: those exhibiting angles between 55° and 125° (rotational mechanism) and those between 145° and 180° (inversion mechanism), across different excitation windows and environments.

Figure 7 presents a schematic overview of the reactive dynamics involved in the photoisomerization of TAE. This figure evidences a correlation between the cis isomer formation and the reaction pathway. Notably, the rotational mechanism tends to favor the formation of cis I, while the inversion mechanism preferentially leads to cis II. In water, the inversion mechanism predominates for both excitation windows, which may be attributed to the higher viscosity of the medium. This increased viscosity can hinder the rotation of the phenyl ring, thus favoring the inversion pathway for isomerization.

**Figure 8:**
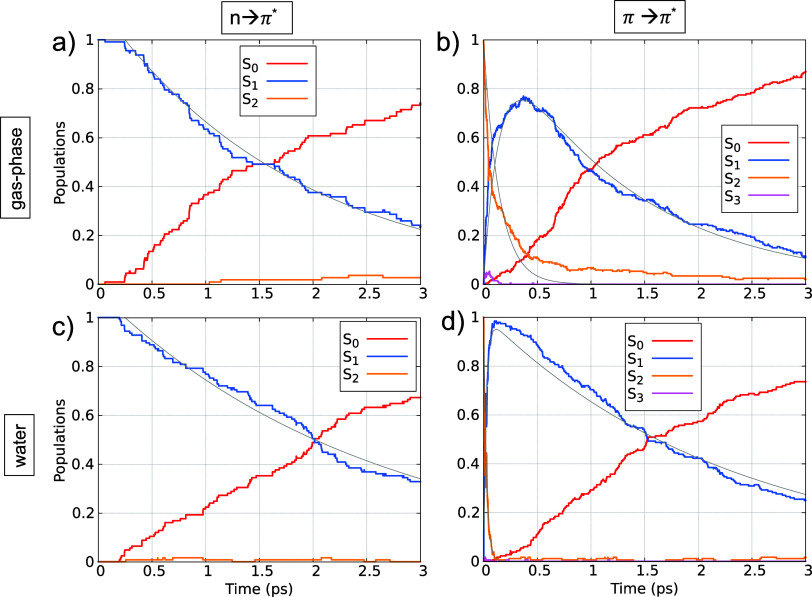
Time-resolved adiabatic state populations of TAE upon excitation to the *n* → π* (panels a and c) and π → π* (panels b and d) states in gas-phase (top) and water (down). Thin gray lines represent population fits. For excitation to the *n* → π* state, the *S*_1_ population is fitted according to eq 1; for the π → π*, the *S*_1_ and *S*_2_ + *S*_3_ populations are fitted according to eq 2.

The population dynamics of the adiabatic states averaged over all trajectories in gas-phase and water up to 3 ps is compared in Figure 8 (see Figure S12 for the full 6 ps propagation in gas-phase). The dynamics starting from the *n* → π* excitation is shown in panels a,b. Initially, all the population resides in the *S*_1_ state. After approximately 250 fs, an exponential decay to the ground state is observed. The *S*_1_ lifetimes, as obtained from eq 1, are summarized in Table 2. The *S*_1_ lifetime is predicted to be longer in water compared to gas-phase, as expected from the higher viscosity and the presence of hydrogen bonds in the aqueous environment, which create additional steric hindrance for the system compared to the gas-phase.

The calculated *S*_1_ state lifetime for TAE is longer than those reported for the parent TAB.^18,26,29^ In the gas-phase, the *S*_1_ state lifetime we obtained for TAE is, in some cases, approximately three times longer than those found in earlier studies of TAB^26,29^ that employed similar methodologies to ours. The presence of the escitalopram group on one side of the molecule, in place of the phenyl ring found in azobenzene, significantly slows down the torsional motion required to reach the region of strong nonadiabatic coupling. This effect is purely kinetic: the increased mass of the escitalopram moiety reduces the rate of torsional rotations, thereby influencing the speed at which the system can transition between states. Additionally, the potential energy curve of the *S*_1_ state for TAE is notably flatter (see Figure 6) compared to the slightly steeper *S*_1_ potential energy curve observed for TAB in previous studies.^26,32,82^ The steeper curve in TAB likely facilitates faster nonadiabatic transitions, contributing to the shorter *S*_1_ state lifetime in TAB relative to TAE. Both the kinetic hindrance introduced by the escitalopram group and the flatter potential energy curve in TAE combine to extend the *S*_1_ state lifetime compared to TAB.

The population dynamics upon excitation to the π → π* states is shown in Figure 8b,d, for gas-phase and water, respectively. At time zero, the population is in the *S*_2_ state (with π → π* character) and quickly decays to the *S*_1_ state (with *n* → π* character), followed by a decay to the ground state. Notably, the *S*_2_ lifetime in water is significantly shorter than in the gas-phase. This behavior can be attributed to the smaller energy difference between the *S*_2_ and *S*_1_ states observed in the absorption spectra for water compared to gas-phase (recall Figure 4). Conversely, the *S*_1_ lifetime is considerably longer in water than in the gas-phase, a trend also seen with *n* → π* excitation.

**Figure 9:**
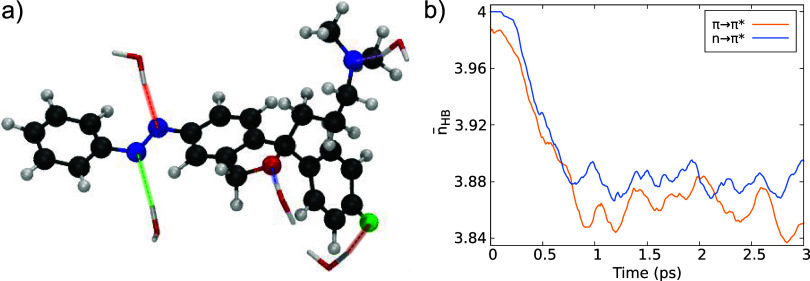
(a) Hydrogen bonds formed by TAE and water after equilibration of the ground state dynamics. (b) Average number of hydrogen bonds between water and the heteroatoms (except for fluorine) of TAE during the excited state dynamics of TAE in water. Results from the *n* → π* excitation in blue and from the π → π* excitation in orange.

We also found it interesting to make an explicit analysis of the hydrogen bonds, since a visual inspection of the ground state thermalization trajectory reveals the presence of five hydrogen bonds between water and TAE, displayed in Figure 9a. There are three hydrogen bonds between the nitrogens of TAE (two nitrogens of the azobenzene moiety and the nitrogen of the amine group) with hydrogens of water and one hydrogen bond between the oxygen of the ether group of TAE with hydrogens of water, which remain more or less stable after equilibration in the ground state dynamics (Figure S6). In contrast, the hydrogen bond between the fluorine of TAE and the hydrogen of water is less stable.

During the excited-state dynamics, the average bond distances of the five hydrogen bonds shown in Figure 9a indicate that the hydrogen bonds involving the oxygen and the nitrogen of the amine group persist over time. In contrast, the hydrogen bonds formed by the nitrogens of the azobenzene moiety and the fluorine gradually dissipate (Figure S13). This behavior is primarily due to the limited movement of the escitalopram moiety, which, owing to its larger mass, exhibits minimal displacement during the dynamics. As a result, only the hydrogen bonds involving the oxygen and the nitrogen of the amine group remain stable over time. However, the photoisomerization mechanism is predominantly driven by the inversion of the nitrogens in the azobenzene moiety and the rotation of the phenyl ring. Consequently, the hydrogen bonds of the nitrogens of the azo group with water molecules can change as the water molecules involved in bonding are exchanged during the dynamics.

The average number of hydrogen bonds formed between TAE and the surrounding water molecules was calculated for all excited-state trajectories (Figure 9b). A hydrogen bond is formed if the distance between the heteroatoms of TAE and the hydrogen atoms of nearby water molecules displaces a maximum threshold of 3.0 Å. Additionally, the angle between the heteroatom of TAE and the water molecule was calculated for all pairs within the distance threshold. A hydrogen bond was considered valid only if the angle exceeded 120°. In order not to have double counting of hydrogen bonds (as water molecules interacting with the nitrogen atoms of the azobenzene group were found to simultaneously fall below both the distance and angle thresholds for both nitrogen atoms), each hydrogen is allowed to form only a single hydrogen bond.

As we can see from Figure 9b, there is a reduction of 0.12 hydrogen bonds for the *n* → π* excitation and a reduction of 0.14 hydrogen bonds for the π → π* excitation after 0.8 ps. After 1.5 ps the average number of hydrogen bonds stabilizes. Fluorine has been excluded from the counting, as its number of averaged hydrogen bonds oscillates too strongly over time. Separate plots of the average number of hydrogen bonds of the two nitrogens of the azobenzene group and the oxygen of the ether together with the nitrogen of the amine group are shown in Figure S14. It is observed that the decrease in the total number of hydrogen bonds is primarily associated with the nitrogens of the azobenzene group. A comparison between the total number of hydrogen bonds (Figure 9b) and those specifically involving the nitrogens of the azobenzene moiety (Figure S14) indicates that the reduction is predominantly occurring in the azo group.

**Figure 10:**
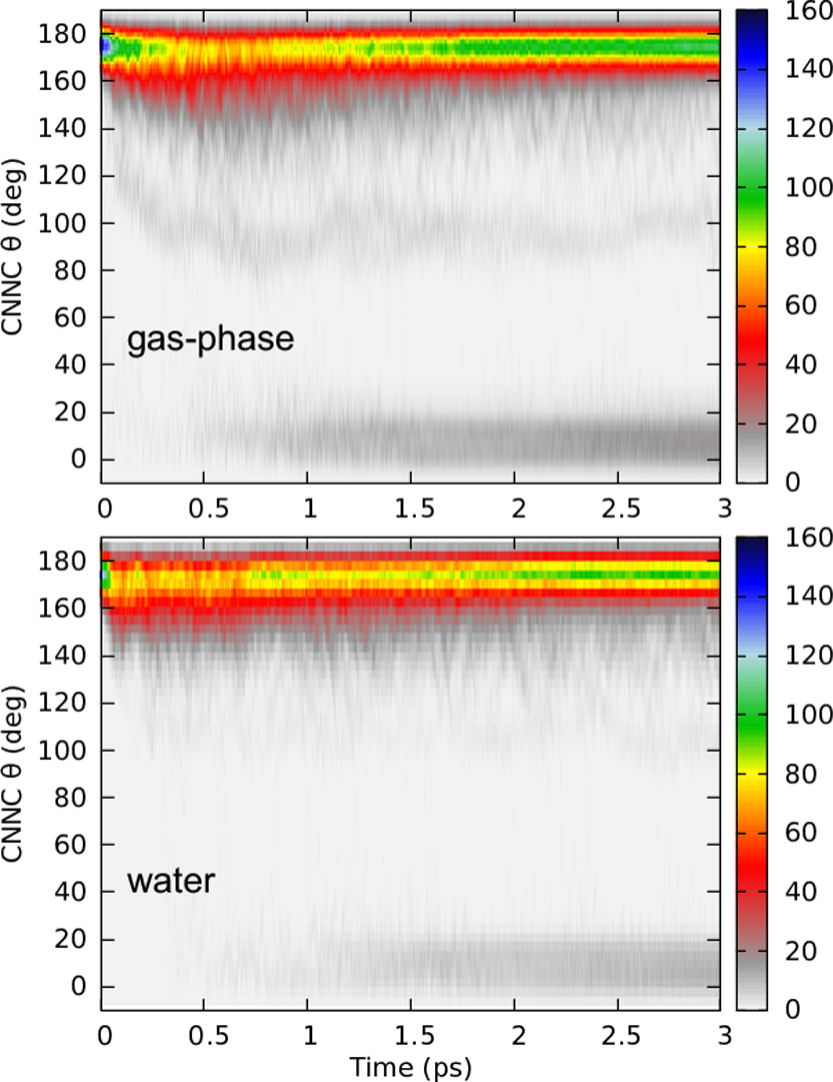
Convolution of the CNNC dihedral over time after π → π* excitation from 207 trajectories in gas-phase (top) and 185 trajectories in water (bottom).

We now turn to analyze the nuclear changes in the solute during the excited state dynamics. The convolution of the CNNC dihedral over time for the trajectories starting in the π → π* state for both gas-phase and water is shown in Figure 10 (the results for the *n* → π* state are presented in Figure S15, and the full 6 ps of the gas-phase trajectories are shown in Figure S16). Initially, all trajectories are in the trans configuration, which is characterized by a dihedral angle between 160 and 180°. As time progresses, we observe fluctuations around the average dihedral angle, with a noticeable shift occurring after approximately 0.5 ps. At this point, geometries with dihedral angles between 0 and 20° begin to emerge, indicating the formation of the cis-conformer. This transition is significantly delayed in the case of water, as expected from the slower *S*_1_ lifetime observed in water. Additionally, a subset of trajectories fluctuates around 100°. Those are the trajectories that they remain in the first excited state since the minimum of the *S*_1_ potential energy curve occurs when the CNNC dihedral is about 100° (see Figure 6).

Likewise, the top panels of Figures S17 (gas-phase) and S18 (water) display the convolution of the CNN angles (γ) as a function of time, while the bottom panels of the same figures present the convolution of the NNC angles (γ′) as a function of time. When the angles approach approximately 140°, it corresponds to geometries close to the crossing point (see Figure 7). As the angles decrease further to around 120°, it indicates that the system decays to the ground state, either in the cis or trans configuration.

## Conclusions

The photoisomerization dynamics of trans-azo-escitalopram, a synthetic photoswitchable inhibitor of the human serotonin transporter, has been investigated both in gas-phase and in explicit water solution. To this aim, we developed an interface between the SHARC package and the MOPAC-PI program, enabling nonadiabatic trajectory surface hopping dynamics simulations using the semiempirical FOMO–CI method on-the-fly, also within a QM/MM framework.

Two excitation windows have been employed: one at lower energy that corresponds to the *n* → π* state and another higher in energy that populates brighter π → π* states. The results indicate that the photoisomerization quantum yield of trans-azo-escitalopram depends on both the excitation window and the environment. Specifically, the quantum yield for the *n* → π* excitation is notably higher than for the π → π* excitation—in agreement with studies on trans-azobenzene.^18^ The surrounding environment also influences the photodynamics, with water significantly reducing the quantum yield relative to gas-phase simulations. Additionally, the *S*_1_ state exhibits a longer lifetime in water than in the gas-phase.

The analysis of the trajectories indicate that most nonadiabatic transitions in trans-azo-escitalopram occur at CNNC dihedral angles between 90° and 120°, also reminiscent to trans-azobenzene.^29^ However, the presence of the escitalopram moiety introduces longer excited-state lifetimes and slower torsional motion, particularly in the *S*_1_ state. A substantial difference from the parent trans-azobenzene is that in trans-azo-escitalopram two distinct cis-isomers are formed during photoisomerization, with a preference for specific isomers depending on the excitation window and solvent environment. In particular, we observed a trend in the formation of a specific isomer depending on whether the photoisomerization proceeds via the rotational or inversion pathway.

Finally, we note that in our simulations the total energy was consistently conserved throughout the dynamics, highlighting the robustness and reliability of the SHARC-semiempirical FOMO–CI implementation for accurately modeling the dynamics of photochemical processes in complex environments. Further studies will investigate the influence of the serotonin transporter on the photoswitching process of azo-escitalopram.

## Supporting Information Available

Molecular orbitals of the active space; ground state thermal dynamics obtained from thermalization trajectory; total energy during excited state dynamics; numerical stability with different time steps; experimental absorption spectrum; population analysis for the gas-phase trajectories; population analysis for the gas-phase trajectories; average hydrogen-bond distance during excited state dynamics; convolution of the CNNC dihedral angle during the excited state dynamics: convolution of the CNN/NNC angles during the excited state dynamics; and CNNC versus CNN/NNC angles for the *S*_1_ → *S*_0_ hopping geometries (PDF)
