# Supplementary material for: Photoisomerization Dynamics of Azo-Escitalopram Using Surface Hopping and a Semiempirical Method
Source: J Phys Chem B. 2024 Dec 21;129(1):385–97. doi: 10.1021/acs.jpcb.4c06924 (PMC11726678; doi:10.1021/acs.jpcb.4c06924)
Supplement: Supplementary file 1 — jp4c06924_si_001.pdf [file jp4c06924_si_001.pdf]

Supplementary Information for:

"Photoisomerization Dynamics of Azo-Escitalopram Using Surface Hopping and a Semiempirical Method"

Hans Georg Gallmetzer[†,‡], Eduarda Sangiogo Gil[‡,a], and Leticia González[¶,b]

†*Doctoral School in Chemistry (DoSChem), University of Vienna, Währinger Str. 42, 1090 Vienna, Austria*

‡*Institute of Theoretical Chemistry, Faculty of Chemistry, University of Vienna, Währinger Str. 17, 1090 Vienna, Austria*

¶*Vienna Research Platform in Accelerating Photoreaction Discovery, University of Vienna, Währinger Str. 17, 1090 Vienna, Austria*

Contents

S1 Molecular orbitals of the active space **S2**

S2 Ground state thermal dynamics **S2**

S3 Total energy during excited state dynamics **S5**

S4 Numerical stability with different time steps **S6**

S5 Experimental absorption spectra **S7**

S6 Population analysis for the gas-phase trajectories **S7**

S7 Average hydrogen-Bond distance and average number of hydrogen-bonds during excited state dynamics **S8**

S8 Convolution of the CNNC dihedral angle during the excited state dynamics **S9**

S9 Convolution of the CNN/NNC angles during the excited state dynamics **S10**

S10CNNC *versus* CNN/NNC angles for the $S_1 \rightarrow S_0$ hopping geometries **S12**

[a] eduarda.sangiogo.gil@univie.ac.at

[b] leticia.gonzalez@univie.ac.at

S1 Molecular orbitals of the active space

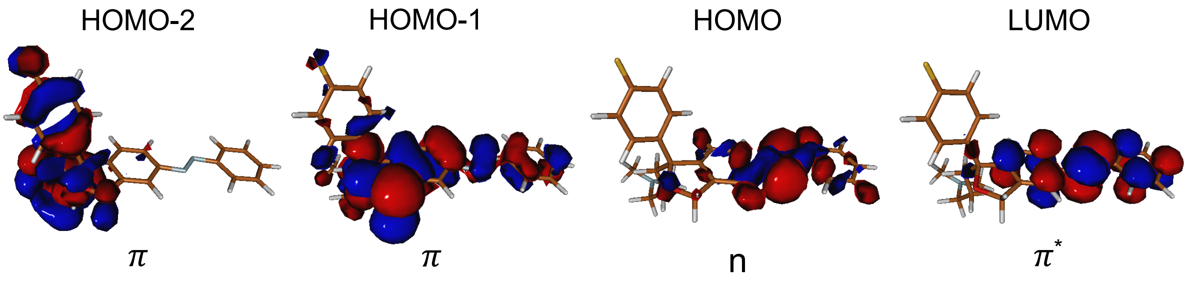

Figure S1: Molecular orbtials of the (6/4) active space of TAE.

S2 Ground state thermal dynamics

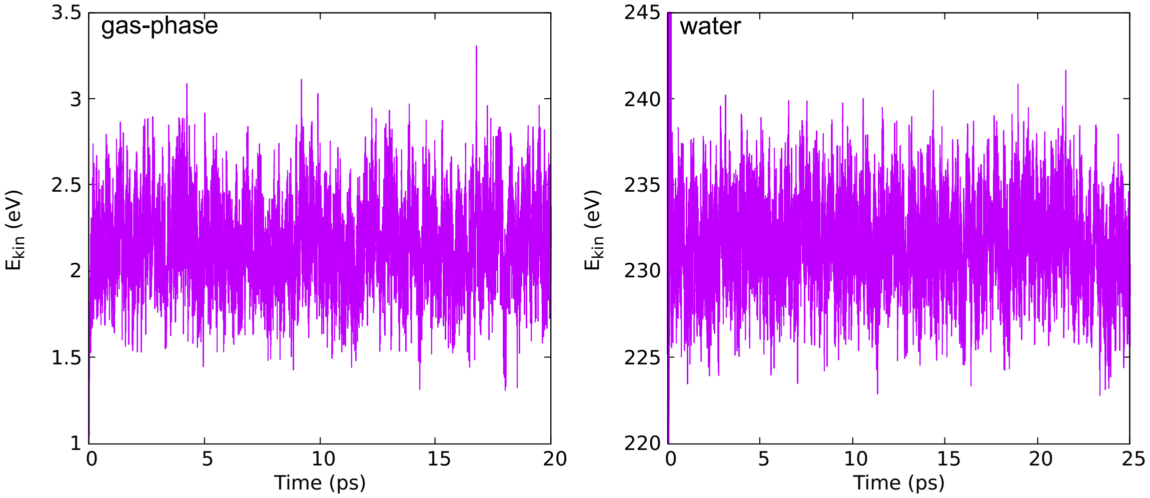

Figure S2: Kinetic energy during thermalization in gas-phase (left) and water (right).

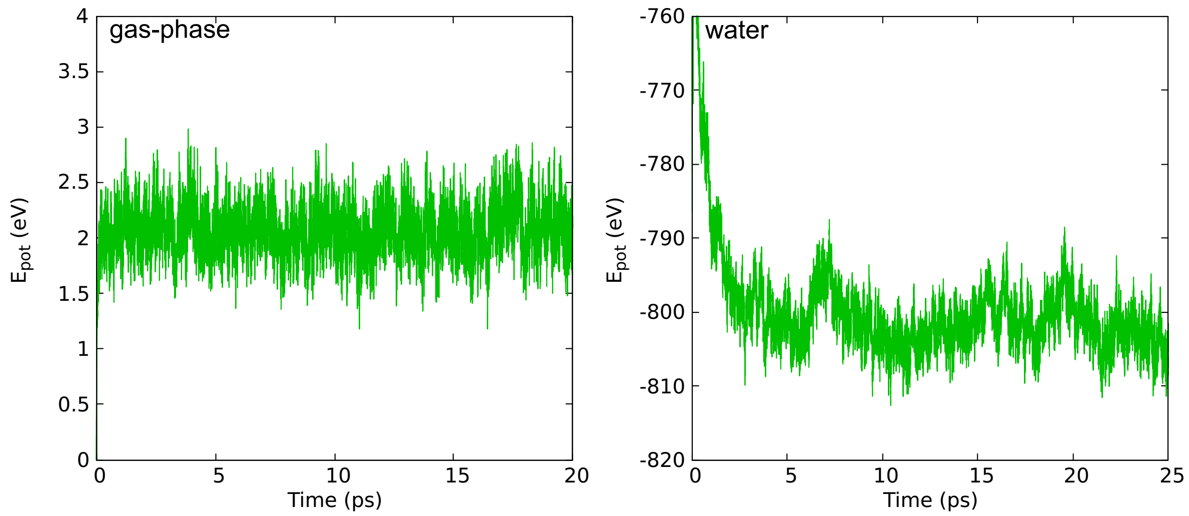

Figure S3: Ground state potential energy relative to the initial potential energy during thermalization in gas-phase (left) and water (right). ($E_{pot} = E_{pot}(t) - E_{pot}(t_0)$)).

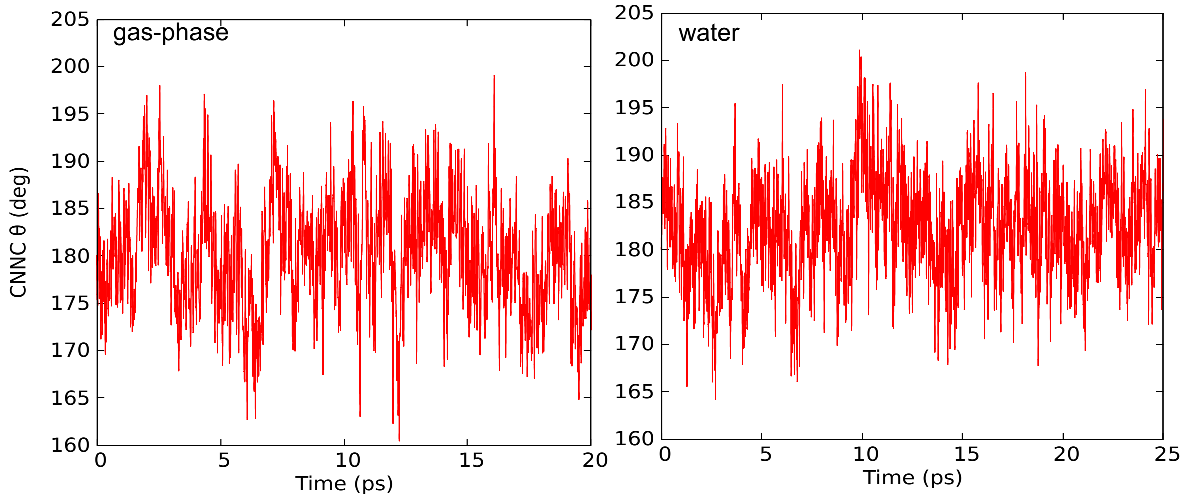

Figure S4: CNNC dihedral (θ) angles during thermalization in gas-phase (left) and water (right).

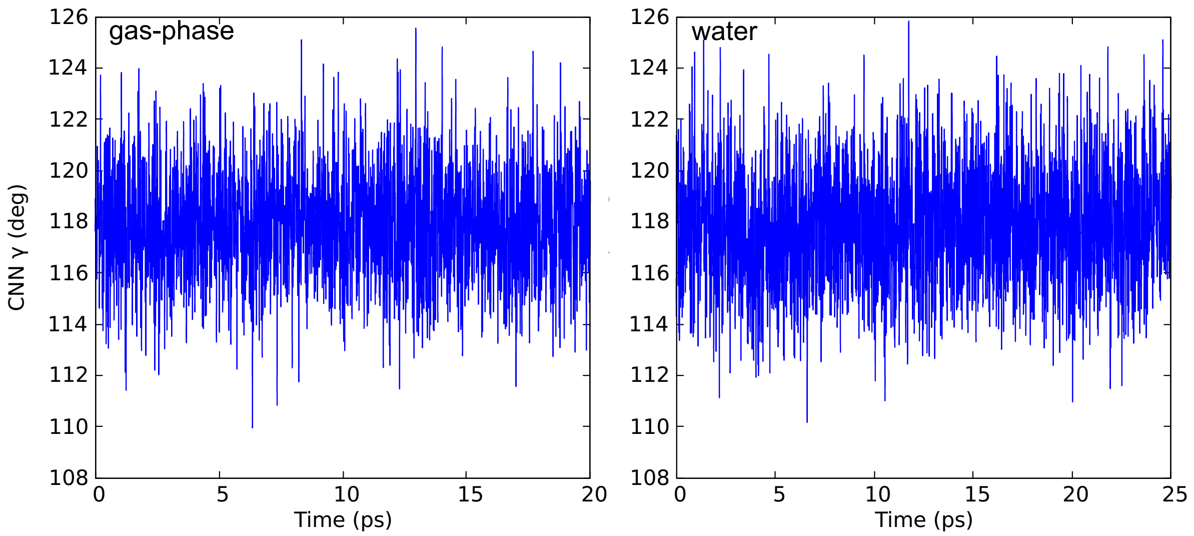

Figure S5: Average between CNN (γ) and NNC (γ') angles during thermalization in gas-phase (left) and water (right).

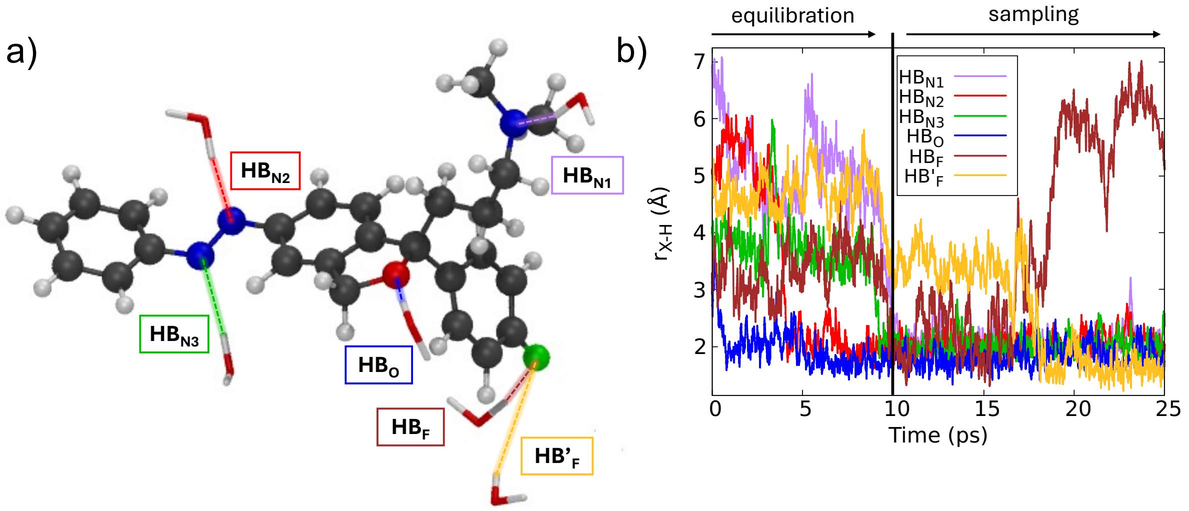

Figure S6: Hydrogen bond distance between TAE and water during the ground state dynamics. Two waters form a hydrogen bond with the flourine of TAE. The system was equilibrated for 10 ps. The remaining 15 ps were used to generate the initial conditions for the excited state dynamics.

S3 Total energy during excited state dynamics

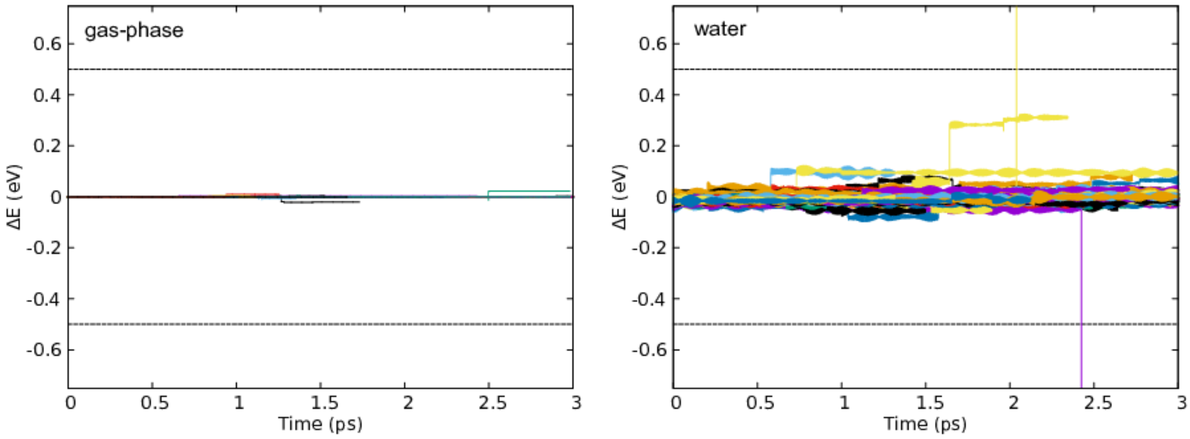

Figure S7: Total energy difference ($\Delta E = E(t) - E(t_0)$) for all individual trajectories (represented by different colors) following $n \rightarrow \pi^*$ excitation, in the gas phase (left) and in water (right). The dashed lines serve as a guide, crossing the y-axis at ± 0.5 eV, which was set as the threshold to exclude trajectories that exceed this limit.

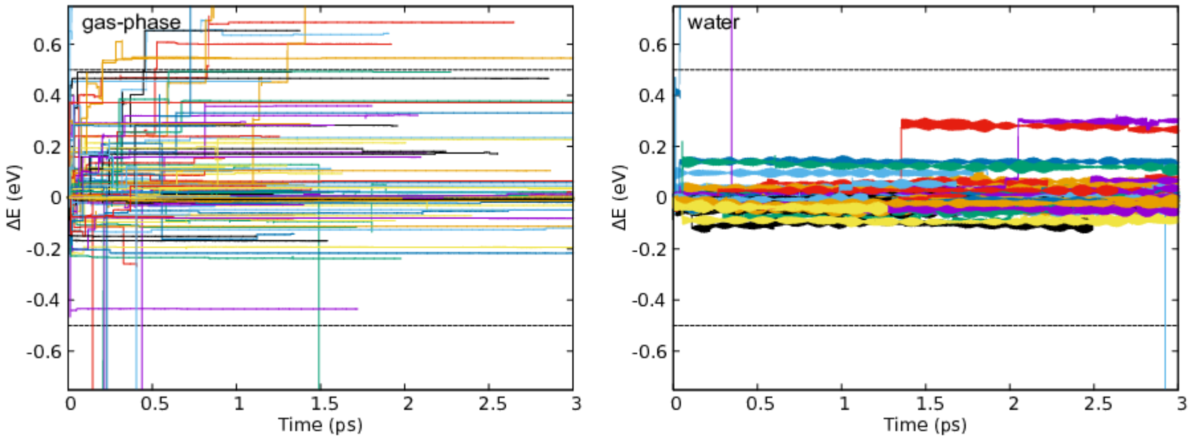

Figure S8: Total energy difference ($\Delta E = E(t) - E(t_0)$) for all individual trajectories (represented by different colors) following $\pi \rightarrow \pi^*$ excitation, in the gas phase (left) and in water (right). The dashed lines serve as a guide, crossing the y-axis at ± 0.5 eV, which was set as the threshold to exclude trajectories that exceed this limit.

S4 Numerical stability with different time steps

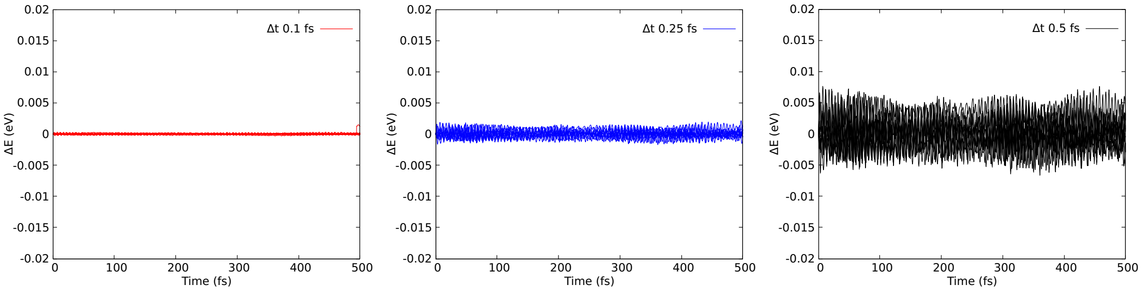

Figure S9: Total energy difference ($\Delta E = E(t) - E(t_0)$) with a time step of 0.1 fs (left), 0.25 fs (middle) and 0.5 fs (right) for a individual trajectory. For each timestep ten trajectories have been run. As can be seen from the plots, bigger time steps have larger fluctuations (as could be expected), but remain numerically stable.

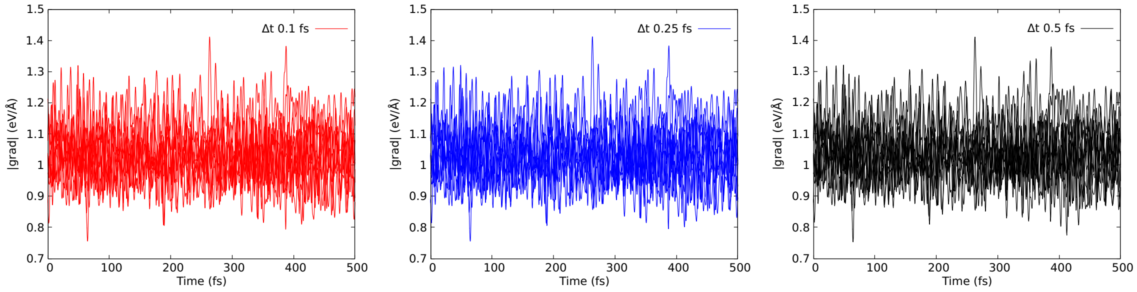

Figure S10: Absolute values of gradients with a time step of 0.1 fs (left), 0.25 fs (middle) and 0.5 fs (right). For each time step ten trajectories have been run.

S5 Experimental absorption spectra

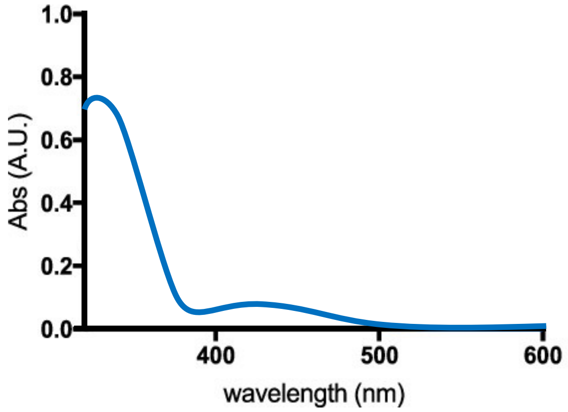

Figure S11: Absorption spectrum of TAE. Adapted from [8]. Copyright [2020] American Chemical Society.

S6 Population analysis for the gas-phase trajectories

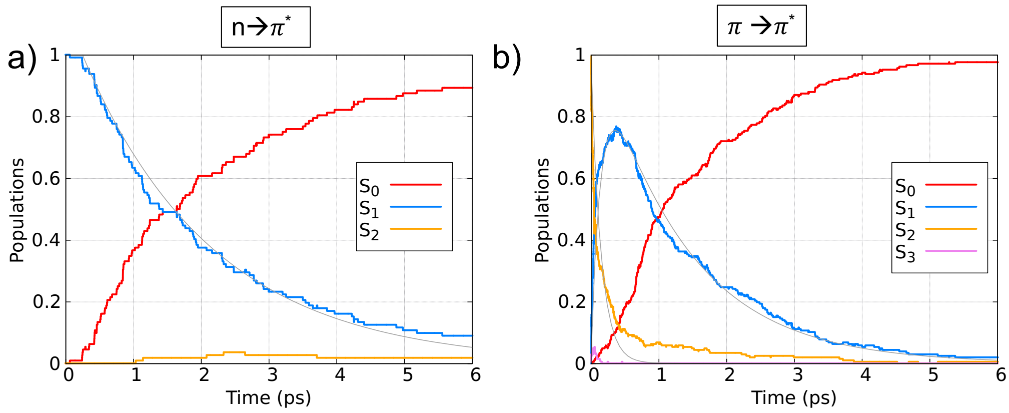

Figure S12: Time-resolved adiabatic state populations of TAE upon excitation to the $n \to \pi^*$ (panel a) and $\pi \to \pi^*$ (panel b) states in gas-phase. Thin gray lines represent population fits.

S7 Average hydrogen-Bond distance and average number of hydrogen-bonds during excited state dynamics

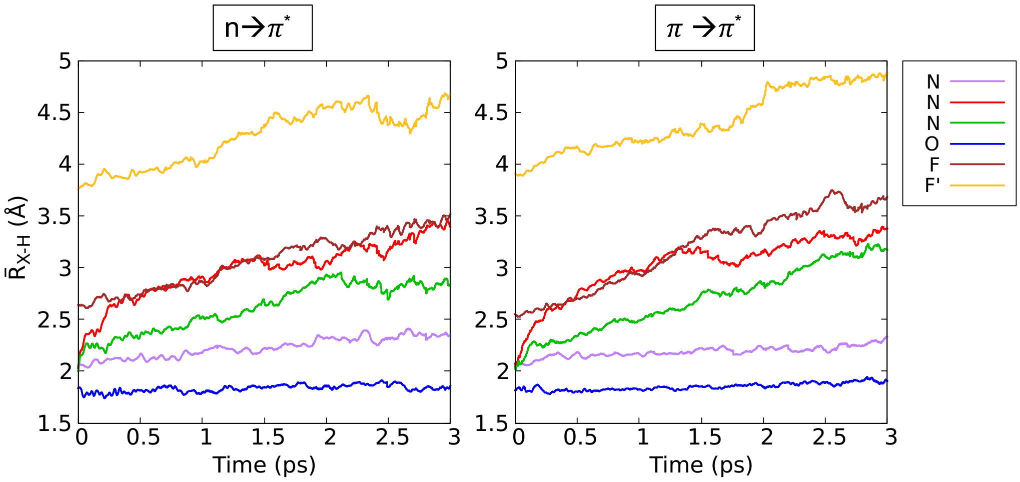

Figure S13: Average hydrogen-bond distance for the six observed hydrogen bonds during the excited state dynamics of TAE in water. Left the $n \rightarrow \pi^*$ excitation and right the $\pi \rightarrow \pi^*$ excitation.

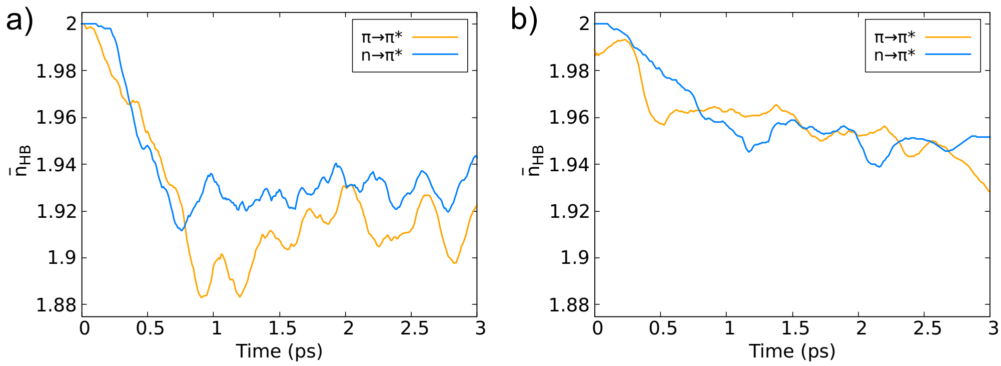

Figure S14: Average number of hydrogen-bonds between water and the hetero-atoms of TAE. a) Hydrogen-bonds of the nitrogens of the azobenzene moiety. b) Hydrogen-bonds of the oxygen of the ether group and the nitrogen of the amine moiety.

S8 Convolution of the CNNC dihedral angle during the excited state dynamics

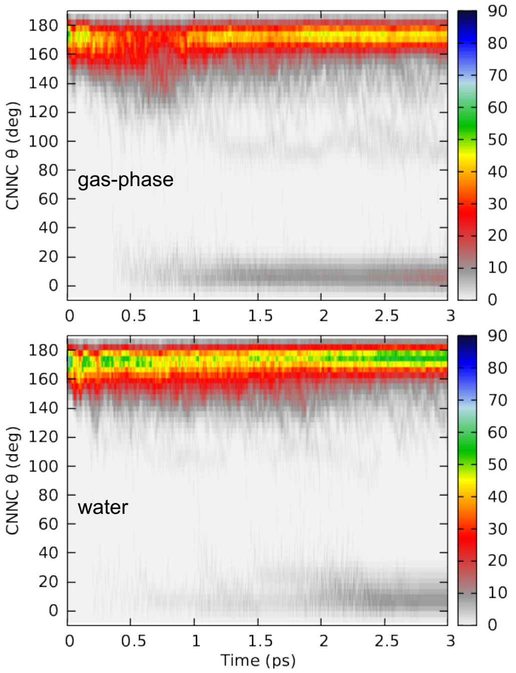

Figure S15: Convolution of the CNNC dihedral over time after $n \rightarrow \pi^*$ excitation from 112 trajectories in gas-phase (top) and 125 trajectories in water (bottom).

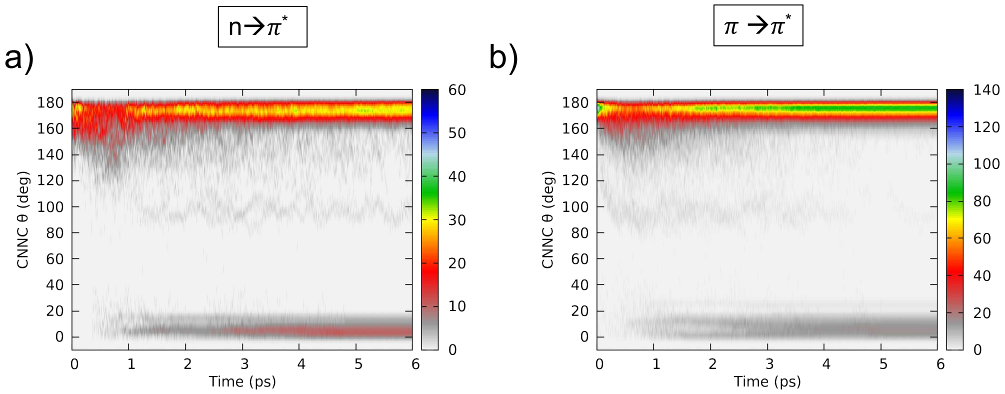

Figure S16: Convolution of the CNNC dihedral over 6 ps after $n \rightarrow \pi^*$ excitation (panel a) from 112 trajectories and after $\pi \rightarrow \pi^*$ excitation (panel b) from 208 trajectories in gas-phase.

S9 Convolution of the CNN/NNC angles during the excited state dynamics

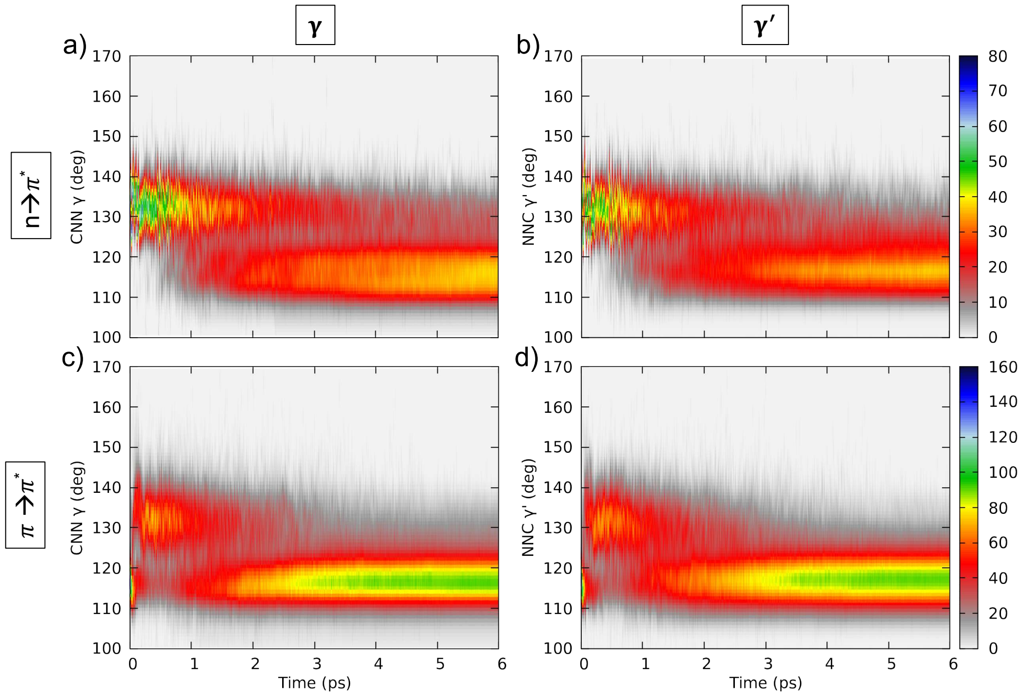

Figure S17: Convolution of the CNN (γ) and NNC (γ') angles over time in gas-phase. a) CNN (γ) after $n \rightarrow \pi^*$ excitation from 112 trajectories. b) NNC (γ') after $n \rightarrow \pi^*$ excitation from 112 trajectories. c) CNN (γ) after $\pi \rightarrow \pi^*$ excitation from 207 trajectories. c) NNC (γ') after $\pi \rightarrow \pi^*$ excitation from 207 trajectories.

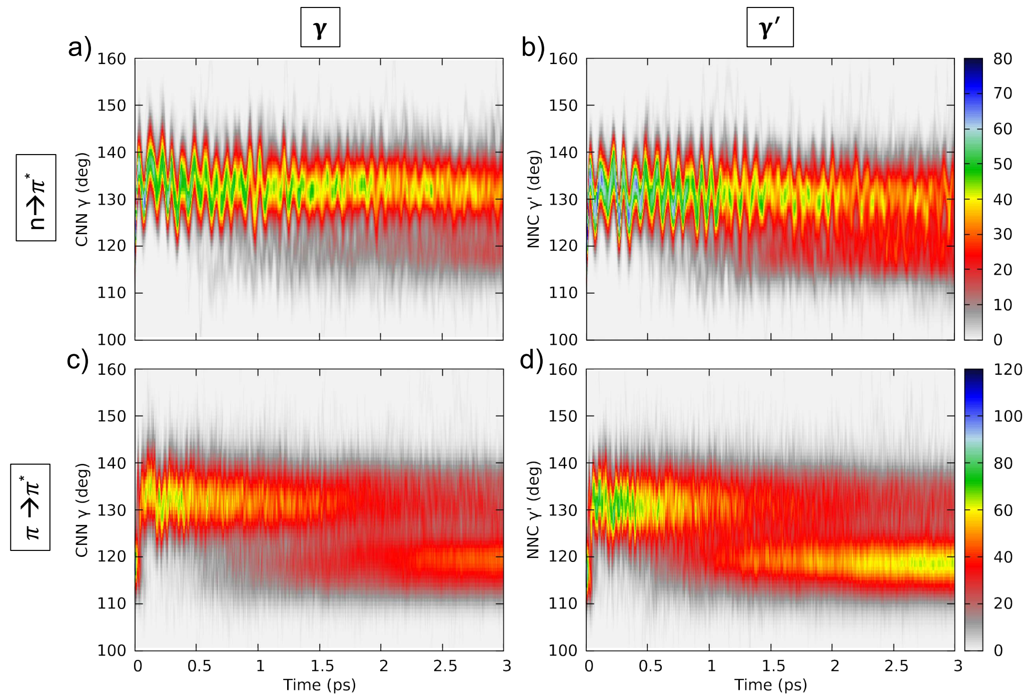

Figure S18: Convolution of CNN (γ) and NNC (γ') angles over time in water. a) CNN (γ) after $n \rightarrow \pi^*$ excitation from 125 trajectories. b) NNC (γ') after $n \rightarrow \pi^*$ excitation from 125 trajectories. c) CNN (γ) after $\pi \rightarrow \pi^*$ excitation from 185 trajectories. c) NNC (γ') after $\pi \rightarrow \pi^*$ excitation from 185 trajectories.

S10 CNNC *versus* CNN/NNC angles for the $S_1 \rightarrow S_0$ hopping geometries

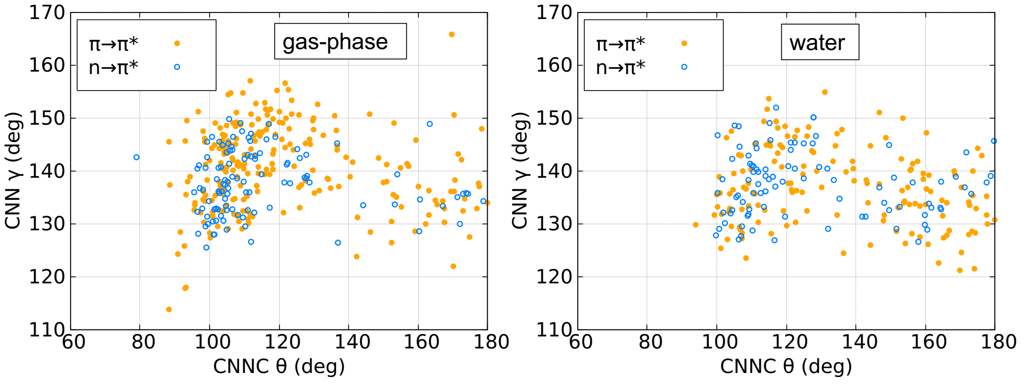

Figure S19: CNNC dihedral angle *versus* CNN angle for the $S_1 \rightarrow S_0$ hopping geometries in gas-phase (left) and water (right). The $\pi \rightarrow \pi^*$ excited trajectories are shown in orange, and $n \rightarrow \pi^*$ excited trajectories in blue.

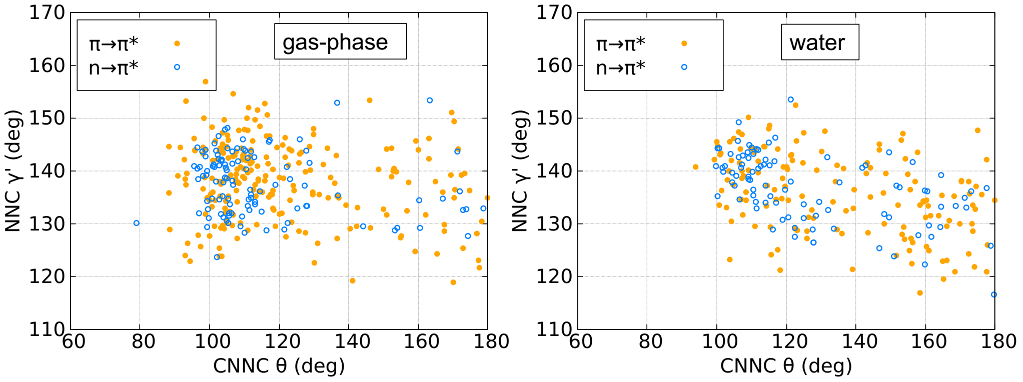

Figure S20: CNNC dihedral angle *versus* NNC angle for the $S_1 \rightarrow S_0$ hopping geometries in gas-phase (left) and water (right). The $\pi \rightarrow \pi^*$ excited trajectories are shown in orange, and $n \rightarrow \pi^*$ excited trajectories in blue.
